# IL-11 system participates in pulmonary artery remodeling and hypertension in pulmonary fibrosis

**DOI:** 10.1186/s12931-022-02241-0

**Published:** 2022-11-15

**Authors:** Javier Milara, Inés Roger, Paula Montero, Enrique Artigues, Juan Escrivá, Julio Cortijo

**Affiliations:** 1 CIBER de Enfermedades Respiratorias, Health Institute Carlos III, Valencia, Spain; 2 Department of Pharmacology, Faculty of Medicine, University of Valencia, Valencia, Spain; 3 Pharmacy Unit, University General Hospital Consortium of Valencia, Valencia, Spain; 4 Surgery Unit, University General Hospital Consortium, Valencia, Spain; 5 Thoracic Surgery Unit, University and Polytechnic Hospital La Fe, Valencia, Spain; 6 Research and Teaching Unit, University General Hospital Consortium, Valencia, Spain; 7 Servicio de Farmacia, Hospital Clínico Universitario de Valencia, Av. de Blasco Ibáñez, 17, 46010 València, Spain

**Keywords:** IL-11, IL-11Rα, Pulmonary hypertension, Pulmonary fibrosis, Pulmonary artery endothelial cells, Pulmonary artery smooth muscle cells

## Abstract

**Background:**

Pulmonary hypertension (PH) associated to idiopathic pulmonary fibrosis (IPF) portends a poor prognosis. IL-11 has been implicated in fibrotic diseases, but their role on pulmonary vessels is unknown. Here we analyzed the contribution of IL-11 to PH in patients with IPF and the potential mechanism implicated.

**Methods:**

Pulmonary arteries, lung tissue and serum of control subjects (n = 20), IPF (n = 20) and PH associated to IPF (n = 20) were used to study the expression and localization of IL-11 and IL-11Rα. Two models of IL-11 and bleomycin-induced lung fibrosis associated to PH were used in Tie2-GFP transgenic mice to evaluate the contribution of IL-11 and endothelial cells to pulmonary artery remodeling. The effect of IL-11 and soluble IL-11Rα on human pulmonary artery endothelial cells and smooth muscle cell transformations and proliferation were analyzed.

**Results:**

IL-11 and IL-11Rα were over-expressed in pulmonary arteries and serum of patients with PH associated to IPF vs IPF patients without PH. Recombinant mice (rm)IL-11 induced lung fibrosis and PH in Tie2-GFP mice, activating in vivo EnMT as a contributor of pulmonary artery remodeling and lung fibrosis. Transient transfection of siRNA-IL-11 reduced lung fibrosis and PH in Tie2-GFP bleomycin model. Human (h)rIL-11 and soluble hrIL-11Rα induced endothelial to mesenchymal transition (EnMT) and pulmonary artery smooth muscle cell to myofibroblast-like transformation, cell proliferation and senescence in vitro.

**Conclusions:**

IL-11 and IL-11Rα are overexpressed in pulmonary arteries of PH associated to IPF patients, and contributes to pulmonary artery remodeling and PH.

**Supplementary Information:**

The online version contains supplementary material available at 10.1186/s12931-022-02241-0.

## Background

Pulmonary hypertension (PH) in chronic lung disease (CLD), such as idiopathic pulmonary fibrosis (IPF) portends a poor prognosis, impairing the median survival time to 2.5–5 years after diagnosis [1]. Recent epidemiological studies reported a 32–84% prevalence of PH in patients with IPF, and PH seems to develop over time in most patients with IPF [2].

The pathobiology of PH in IPF is incompletely understood. While the classical hypothesis of PH development in IPF was the parenchymal fibrotic destruction and hypoxic vasoconstriction, recent evidence indicate that pulmonary artery remodeling can drive the increase of pulmonary artery tension, since no correlation exist between lung fibrosis severity and PH development [3]. Biological processes underlying fibrosis progression are also involved in the vascular remodeling and PH [3]. In this regard, pulmonary vascular muscularisation in the early stages followed by fibrous vascular atrophy and pronounced intimal fibrosis are described [4]. Blood vessels may participate in the genesis of IPF, and similar molecular disruptions have been described in patients with IPF and PH [5]. The latter suggests that these disorders share pathogenic features and that one may influence, generate, or perpetuate the other. Different cellular processes have been described during pulmonary artery (PA) remodelling of PH-associated IPF, including endothelial dysfunction [6], the endothelial-to-mesenchymal transition (EnMT) as a source of myofibroblasts [7, 8], as well as PA smooth muscle proliferation and transition to myofibroblast-like phenotype [9].

Despite the major role of PH in IPF patient survival, no clinically approved drugs yet exist to treat PH in IPF patients, since classical vasodilators used to treat PAH, such as phosphodiesterase 5 inhibitors, endothelin receptor inhibitors [10] or guanylate cyclase stimulator [11] yielded results that were inconclusive at best [12]. The lack of approved drugs for PH in PF patients highlights the need for a better understanding of the pathophysiology and molecular mechanisms underlying PH in PF.

Recent findings have highlighted the role of IL-11 in different fibrotic diseases including cardiac [13] and pulmonary fibrosis [14]. IL-11 promotes lung fibroblast activation and transformation into myofibroblasts as well as the alveolar epithelial to mesenchymal transition promoting extracellular matrix accumulation, as well as lung fibrosis when was administered in mice [14]. Both IL-11 and its cognate receptor IL-11Rα are overexpressed in fibrotic areas from IPF patients, and typical pro-fibrotic factors, such as TGFβ1, induce its over-expression [14]. However, the role of IL-11 on vascular function and the participation of the IL-11 system in PH of IPF patients is completely unknown. Here we explored whether IL-11 and IL-11Rα is observed in pulmonary arteries of patients with IPF and PH, and whether IL-11 is linked to pulmonary artery endothelial and smooth muscle cell pathobiology of pulmonary artery remodelling. We then analysed whether IL-11 signalling participates in pulmonary artery remodelling and fibrosis through its activity in pulmonary endothelial and smooth muscle cells. We ended studying the effect of IL-11 inducing pulmonary hypertension and the role of *IL-11* gene silencing as a therapeutic target in different animal models of pulmonary fibrosis and pulmonary hypertension.

## Methods

### Patients

Human lung tissue was obtained from 3 types of patients (Thoracic Surgery and Pathology Services of the University General Consortium Hospital (CHGUV) and University and Polytechnic Hospital La Fe, Spain): (A) Patients with PH-associated IPF who underwent surgery for organ transplantation program. (B) Patients with IPF who underwent surgery for organ transplantation program (represents pulmonary arteries with IPF and without PH). (C) Lung explant control samples were obtained from organ transplant program from CHGUV, in donors with normal lung function that was not used for transplant purposes (represents lung tissue without IPF and were used as controls), without any lung disease (*n* numbers are indicated in every figure).

IPF and PH were diagnosed according to the American Thoracic Society/European Respiratory Society and European Society of Cardiology consensus criteria [15, 16]. All pulmonary function tests were performed within 3 months before surgery. After selection based on diagnosis criteria, all lung tissue samples used for the study were checked histologically by using the following exclusion criteria: (1) presence of tumor, (2) respiratory tract infection. The lungs taken from donor controls showed normal architecture with few intra-alveolar macrophages and edema. Muscular pulmonary arteries (70–500 μm diameter) and whole lung tissue homogenates were isolated from lungs to compare molecular expression from lung parenchyma and between patients. Serum from peripheral venous blood was isolated to determine IL-11 and circulating IL-11Rα serum proteins. Patients included in this work were free of auto-immune co-morbidities and did not use immunomodulatory drugs. Clinical patient characteristics are shown in Additional file 8: Tables S1–S3. The protocol was approved by the local research and independent ethics committee of the University General Consortium Hospital of Valencia (CEIC22/2017). Informed written consent was obtained from each participant.

### Histological, immunohistochemical and immunofluorescence studies

Lung histology was conducted as previously reported [17]. Tissue blocks (4 μm thickness) were stained with Masson’s trichrome (Sigma-Aldrich, Madrid, Spain) to detect collagen deposition and for assessment of the fibrotic injury and pulmonary artery remodeling. Severity of lung fibrosis was scored on a scale from 0 (normal lung) to 8 (total fibrotic obliteration of fields) according to Ashcroft score [18].

For immunohistochemical analysis of human lungs, tissue was fixed and embedded in paraffin, cut into sections (4–6 µm) and incubated with IL-11 (Thermo Fisher; catalogue no. PA5-36544; dilution 1:100), IL-11RΑ (Novus Biological; catalogue no. NBP1-62351; dilution 1:50) and αSMA (Cell Signaling; catalogue no. 19245S; dilution 1:200) antibodies for 24 h at 4 °C. A secondary anti-rabbit antibody (1:100; Vector Laboratories, Burlingame, CA) with avidin–biotin complex/horseradish peroxidase was used for immunohistochemistry. The non-immune IgG isotype was used as a negative control and yielded no signal in all cases (data not shown). Vascular wall thickness and immunohistochemical composite score were assessed on muscular pulmonary arteries (70–500 µm) using 20–30 pulmonary arteries per patient (“n”) randomly selected from each group. For fibrotic and non-fibrotic areas, vascular wall thickness (WT) was quantified by averaging the external and internal diameter of transversally cut vessels and determining the mean distance between the lamina elastica externa and lumen in two perpendicular directions calculated as WT = (do–di)/do × 100%, with “do” and “di” external and internal diameter as already described [19, 20]. IL-11/IL-11Rα stained slices were scored by a pathologist under a Nikon Eclipse TE200 light microscope (Tokio, Japan), using 20–30 pulmonary arteries per patient (“n”) randomly selected from each group. Staining intensity was analyzed in pulmonary arteries. Staining intensity for different antibodies was scored on a scale of 0 to 3 (0, negative; 1, weak; 2, moderate; 3, strong immunoreactivity). The percentage of cells positive for different antibodies within pulmonary arteries was scored on a scale of 1 to 4 as follows: 1, 0–25% cells positive; 2, 26–50% positive; 3, 51–75% positive; and 4, 76–100% positive. The score of the staining intensity and percentage of immunoreactive cells were then multiplied to obtain a composite score ranging from 0 to 12 as previously outlined [21].

IL-11 (Invitrogen; catalogue no. PA5-36544), IL-11RΑ (Novus Biological; catalogue no. NBP1-62351), αSMA (Cell Signaling; catalogue no. 19245S/ Sigma-Aldrich; catalogue no. A5228), FAP (Invitrogen; catalogue no. PA5-27773), and GFP (Invitrogen; catalogue no. A11122 and A11120) were analyzed by immunofluorescence. Cells and tissue were fixed in paraformaldehyde (4%) for 48 h, and tissue was embedded in Tissue-Tek® OCT™ cryosectioning compound (Sakura Finetek Europe BV, Leiden). Blocks were cut into 10 µm thick sections, permeabilized in Triton X 100 (0.1% in PBS) for 5 min, blocked in 10% goat serum in PBS and immunostained with the above described antibodies for 24 h at 4  C followed by a secondary FITC or rhodamine conjugated anti-mouse/rabbit IgG antibody and finally DAPI (2 µg/ml) to mark nuclei (Molecular Probes, Leiden, The Netherlands). Co-localization of IL-11/αSMA, IL-11RΑ/αSMA, FAP/GFP, and αSMA/GFP was performed using a confocal spectral Leica TCS SP2 microscope with × 1000 magnification and 3 × zoom. Red (HeNe 543 nm), green (HeNe 488 nm), and blue (Ar 351 nm, 364 nm) lasers were used. Co-localization studies were performed using the Leica confocal software v2.61. The cell images with colocalized points of the two laser canals were transformed into a white/orange color in the image.

### Isolation and culture of human pulmonary artery endothelial cells and pulmonary arterial smooth muscle cells and in vitro experimental conditions

Cellular experiments were performed in primary human pulmonary artery endothelial cells (HPAECs) and human pulmonary artery smooth muscle cells (HPASMCs) isolated from lungs of different patients. Segments of pulmonary artery (70–500 μm internal diameter) were dissected free from parenchyma lung tissue, cut longitudinally, and digested with 1% collagenase (Gibco, UK) in RPMI-1640 culture medium for 30 min at 37 °C. The digestion was neutralized by adding RPMI 1640 supplemented with 20% foetal calf serum (FCS), and the homogenate was separated by centrifugation at 150 g. The pellet was resuspended, and cells were cultured in EGM-2 endothelial culture medium supplemented with Single Quotes (Clonetics, UK), 10% FCS, 1% fungizone, and 2% streptomycin/penicillin. The selection of HPAECs was performed as we previously described [22–24], modified to include the use of a commercially available Dynabeads CD31 endothelial cell kit (Dynal Biotech, Germany). Briefly, cells were trypsinized (0.25% trypsin), and the cell mixture was incubated with CD-31-coated Dynabeads for 30 min at 4 °C with end-over-end rotation. After incubation, the HPAECs were collected using a magnetic particle concentrator (MCP-1; Dynal) and washed four times with cold phosphate-buffered saline (PBS)/bovine serum albumin (BSA). Clusters of purified HPAECs retained on the CD-31-coated Dynabeads were separately resuspended in EGM-2 full growth medium supplemented with 10% FCS, 1% fungizone, and 2% streptomycin/penicillin.

The cells not retained on the CD-31-coated Dynabeads, i.e., HPASMCs, were cultured in smooth muscle cell growth medium 2 (Promocell, cat.n. C-22062) supplemented with supplementedMix (Promocell cat. n. C-39267) to selectively separate the HPASMCs. For positive identification of HPASMCs, the cells were subcultured for one passage, and α-actin expression was examined using a monoclonal antibody against α-smooth muscle actin (1:100 dilution; Sigma); > 95% of the cells were positively stained. HPASMC were negative for CD90 (fibroblasts), CD31 and CD144 (endothelial cells). The cells were incubated for 16 h in 0.5% FCS culture medium before each experiment and were returned to 5% FCS culture medium at the start of each experimental condition. Primary cells were used at cell passage ≤ 3.

Mice lung fibroblasts were isolated from whole lung. Parenchyma fragments (approximately 5 × 5 mm) were washed with sterile physiological saline and incubated in culture plates with dulbecco's modified eagle culture medium (DMEM) with 10% serum foetal bovine, 100U/ml Penicillin/Streptomycin and 2.5 μg/ml amphotericin B. Cultures were spared in a humidified incubator with 5% CO_2_ at 37 °C. After a period of approximately 1 week, fibroblasts were observed around the parenchyma fragments. Fibroblast characterization was performed by collagen type I, CD34 + , FAP and αSMA positivity.

For in vitro studies, HPAECs, HPASMCs and mice lung fibroblasts were stimulated with recombinant human IL-11 (rhIL-11, 5 ng/ml; cat. n. SRP3072, Sigma Aldrich), recombinant mice IL-11 (rmIL-11, 5 ng/ml; cat. n. Z03052-1, GeneScript), recombinant human IL-11RΑ (rhIL-11RΑ 10 ng/ml; cat. n. H00003590-P01, NOVUSBIO), recombinant mouse IL-11RΑ (rmIL-11RΑ 10 ng/ml; cat. n. 50,075-M08H, SinoBiological), or combinations for the indicated times, replacing culture medium and stimulus every 24 h. Selected concentrations were from concentration dependent curves on airway epithelial cells (data not shown) and literature [14]. JSI-124 (JAK2 inhibitor; Sigma Aldrich), SIS3 (SMAD3 inhibitor; Sigma Aldrich) or PD98059 (ERK inhibitor; Sigma Aldrich) were added 30 min before stimulus and remained together with the stimulus until experimental evaluation. None of the drugs affected cell viability assessed with trypan blue (Sigma), showing greater than 95% viability. Cellular extracts were collected to analyze the expression of different proteins and genes.

Protein levels of CD31, IL-11, IL-11Rα were measured in isolated human pulmonary arteries, human lung tissue homogenates and serum, using commercially available ELISA kits for CD31 (R&D systems; cat. n. DCD310), IL-11 (Sigma-Aldrich; cat. n. RAB0250) and IL-11RΑ (Mybiosource; cat. n. MBS047882) according with the manufacturer instructions.

### Real-time RT-PCR

Total RNA was isolated using TriPure® Isolation Reagent (Roche, Indianapolis, USA). The integrity of the extracted RNA was confirmed with Bioanalyzer (Agilent, Palo Alto, CA, USA). Reverse transcription was performed in 300 ng of total RNA with a TaqMan reverse transcription reagents kit (Applied Biosystems, Perkin-Elmer Corporation, CA, USA). cDNA was amplified with specific primers and probes predesigned by Applied Biosystems for human: IL-11Rα (Hs01039494_m1), α1(I)-collagen (collagen type I; Hs00164004_m1), ET-1 (Hs00174961_m1), TWIST-1 (Hs00361186_m1), FGF (Hs01040810_m1), VEGF (Hs0090055_m1), PDGF (Hs00966522_m1), vimentin (Hs00958111_m1), αSMA (Hs00559403_m1), VE-cadherin (Hs00901469_m1), FVIII (Hs00252034_m1), NOS3 (Hs01574659_m1), CTGF (Hs00170014_m1), CD31 (Hs01065282_m1), P21 (Hs01040810_m1), P16 (Hs00923894_m1), fibronectin (Hs01549976_m1) and TGFβ1 (Hs00998133_m1); Mouse IL-11 (Mm00434162_m1), IL-11Rα (Mm00494938_m1), α1(I)-collagen (Mm00801666_g1), αSMA (Mm00808218_g1), FGF (Mm01285715_m1), PDGF (Mm00440677_m1), CTGF (Mm00515790_g1), TGFβ1 (Mm00441724_m1), fibronectin (Mm01256744_m1), ET-1 (Mn00438656_m1), FVIII (Mm01215675_m1), CD31 (Mm01242576), NOS3 (Mm00435217_m1), E-cadherin (Mm01247357_m1) and VE-cadherin (Mm00486938) in a 7900HT Fast Real-Time PCR System (Applied Biosystems) using Universal Master Mix (Applied Biosystems). Expression of the target gene was expressed as the fold increase or decrease relative to the expression of β-actin as an endogenous control (Applied Biosystems; Hs01060665 for human and Mm02619580_g1 for mouse). The mean value of the replicates for each sample was calculated and expressed as the cycle threshold (Ct). The level of gene expression was then calculated as the difference (ΔCt) between the Ct value of the target gene and the Ct value of β-actin. The fold changes in the target gene mRNA levels were designated 2^−ΔCt^.

### Western blotting analysis

Western blotting analysis was used to detect changes in human and mouse lung tissues, as well as in HPAECs and HASMCs cell protein expression. Lung tissue or cells were homogenized or scraped from a confluent 25-cm^2^ flask and lysed on ice with a lysis buffer comprising a complete inhibitor cocktail plus 1 mM ethylenediaminetetraacectic acid (Roche Diagnostics Ltd., West Sussex, UK) with 20 mM Tris base, 0.9% NaCl, 0.1% Triton X-100, 1 mM dithiothreitol, and 1 mg/mL pepstatin A. The Bio-Rad assay (Bio-Rad Laboratories Ltd., Herts, UK) was used according to the manufacturer’s instructions to quantify the level of protein in each sample to ensure equal protein loading. Sodium dodecyl sulfate polyacrylamide gel electrophoresis was used to separate the proteins according to their molecular weight. Briefly, 15 µg of proteins (denatured) along with a molecular weight protein marker (Bio-Rad Kaleidoscope marker; Bio-Rad Laboratories) were loaded onto an acrylamide gel consisting of a 5% acrylamide stacking gel stacked on top of a 10% acrylamide resolving gel and run through the gel by application of 100 V for 1 h. Proteins were transferred from the gel to a polyvinylidene difluoride membrane using a wet-blotting method. The membrane was blocked with 5% Marvel in PBS containing 0.1% Tween20 (PBS-T), probed with the following antibodies:

p-GP130 (Invitrogen, cat. n. PA5-64830), GP130 (Cell Signaling, cat. n. 3732S), p-JAK2 (Cell Signaling,, cat. n. 3771), JAK2 (Cell Signaling,, cat. n. 3230), p-STAT3 (Cell Signaling, cat. n. 9145S), STAT3 (Novus Biologicals, cat. n. NB100-91973), p-AKT (Cell Signaling, cat. n. 2965), AKT (Cell Signaling, cat. n. 4685S), p-ERK1/2 (Sigma-Aldrich, cat. n. M-9692), ERK1/2 (Cell Signaling, cat. n. 4695), p-SMAD 2/3 (Millipore, cat. n. PS1023), SMAD 2/3 (Calbiochem, cat. n. 566414), P21 (Novus Biologicals, cat. n. NB100-1941), collagen type 1 (calbiochem, cat. n. 234167), α-SMA (Cell Signaling, cat. n. 19245S), VE-Cadherin (Invitrogen, cat. n. 36-1900), β-actin (Sigma-Aldrich, cat. n. A1978). The enhanced chemiluminescence method of protein detection using enhanced chemiluminescence reagents (ECL Plus; Amersham GE Healthcare, Buckinghamshire, UK) was used to detect labeled proteins. Densitometry of films was performed using the Image J 1.42q software (available at http://rsb.info.nih.gov/ij/, USA). Results of target protein expression are expressed as the ratio of non-phosphorylated form or densitometry of the endogenous controls β-actin.

### Cell senescence and proliferation

Cells were stimulated with IL-11, IL-11RΑ or their combination during 72 h. The senescence cell histochemical staining kit (Sigma Aldrich; catalogue no. CS0030) based on a histochemical staining for β-galactosidase activity was used. After stimulation, cells were fixed with the fixation buffer provided by the kit (solution containing 20% formaldehyde, 2% glutaraldehyde, 70.4 mM Na2HPO4, 14.7 mM KH2PO4, 1.37 M NaCl, and 26.8 mM KCl) for 6–7 min at room temperature. Cells were stained with the staining mixture provided by the kit and incubated at 37 °C without CO_2_ overnight. Finally, cells were observed under a microscope to count the blue-stained cells and the total number of cells x field. Results were expressed as % senescence (β-galactosidase blue positive cells) relative to the total number of cells in each field in a total of 5 fields.

Proliferation was measured by colorimetric immunoassay based on BrdU incorporation during DNA synthesis using a cell proliferation enzyme-linked immunosorbent assay BrdU kit (Roche, Mannheim, Germany) according to the manufacturer’s protocol. Cells were seeded at a density of 3 × 10^3^ cells/well on 96-well plates and incubated for 24 h. Cells were stimulated with IL-11, IL-11Rα or their combination and incubated at indicated times. The 490 nm absorbance was quantified using a microplate spectrophotometer (Victor 1420 Multilabel Counter, PerkinElmer). Proliferation data refer to the absorbance values of BrdU-labeled cellular DNA content per well.

### Pulmonary hypertension animal models

Experimentation and handling were performed in accordance with the guidelines of the Committee of Animal Ethics and Well-being of the University of Valencia (2019/VSC/PEA/0002 and 2019/VSC/PEA/0235 Valencia, Spain) following the ARRIVE guidelines [25]. Animal were housed with free access to water and food under standard conditions: relative humidity 55 ± 10%; temperature 22 ± 3ºC; 15 air cycles/ per hour; 12/12 h Light/Dark cycle. Mice studies used pathogen-free male Tie2-GFP 287Sato/J transgenic male mice (Jackson Laboratory, Bar Harbor, ME, USA) 24 weeks of age and of an approximate weight between 25-28 g. Two different models were performed in Tie-GFP mice; (1) subcutaneous (s.c) IL-11 mice model, (2) Intratracheal (I.T) bleomycin mice model. The account for experimental groups was estimated in a number of 11 mice (n = 11) based in previous findings [14, 26]. The primary outcome was the 30% of differences in right ventricular systolic pressure (RVSP) between treatment groups calculated for an effect size f of 1.8, α error of 0.05 and power of 0.96, using one tailed analysis of variance (ANOVA). Sample size for tissue- and cell-based assays were determined based on sample availability and technical needs. Animals were randomly assigned to experimental groups on the day before treatment. Animal lung tissue analysis, histology and pathological scoring were performed blinded to treatment. No criteria were set to including or excluding animals during the experiments.

In the first animal models, mouse recombinant IL-11 100 μg/kg (n = 11) or saline (n = 11) were daily administered subcutaneously during 21 days developing lung fibrosis as previously outlined [14]. In the second model, the IT bleomycin model was assayed as previously we outlined [27]. Transient IL-11-KO Tie-GFP mice were generated as follow; For in vivo RNAi studies, IL-11 siRNA (ID AM16832, Ambion) or non-targeting control siRNA (Cat#: 4404021, Ambion) were reconstituted (5 µg/µl) in nuclease-free water and administered to the lungs to 24 weeks old mice by intranasal and intravenous delivery. Intranasal delivery was at a dose of 50 µg per treatment in a total volume of 20 µL of PBS. Intravenous delivery was as siRNA/polyethylenimine linear (in vivo-jetPEI®, Polyplus-transfection S.A. Cat. n. 201-50G) complexes. A dose of 40 µg siRNA was diluted into 100 µl of 5% glucose and a dose of 6.4 µl polyethylenimine was diluted into 100 µl of 5% glucose. In both cases 10% glucose stock solution was used. Diluted polyethylenimine was added to the diluted siRNA all at once, vortex gently and spin down. Mix was incubated for 15 min at room temperature and immediately delivered into tail vein. Intranasal and intravenous delivery were repeated during 14 days every 48 h.

24 weeks age male mice were anaesthetized with ketamine/medetomidine and then a single dose of bleomycin at 1.5 U/kg (dissolved in 20µL of saline) was administered IT via the endotracheal route [28]. Sham control treated mice received the identical volume of IT saline instead of bleomycin. This procedure fixed experimentation day 1. The account for experimental groups was estimated in a number of 11 mice (n = 11) per group: The IL-11-KO protocol was designed with the following groups: (i) siRNA(-) vehicle animals; (ii) siRNA-IL-11 vehicle animals (iii) bleomycin siRNA(-) animals; (iv) bleomycin siRNA-IL-11 animals. Results from the siRNA-IL-11-vehicle group were not significantly different from the control-vehicle group and were not represented in the figures.

At the end of the procedures, animals were anesthetized using 5% induction isoflurane and 2% during maintenance. Buprenorfin 0.1 ml/Kg y meloxicam 0.3 ml/Kg were administered intraperitonealy as analgesics in order to perform hemodynamic studies. RVSP was measured by right heart catheterization. The right jugular vein was cannulated with a small silicone catheter (BPE-T50 Polyethylene tubing for 22ga swivels; Salomon Scientific, CA, USA) containing heparin saline solution (10 UI/ml of heparin in 0.9% saline), to reach the RV under the guidance of the pressure tracing. After 20 min of stabilization, RVSP was recorded using a miniature pressure transducer (TSD104A, BIOPAC Systems, Inc., CA, USA) digitized by a BIOPAC MP100 data acquisition system.

After hemodynamic studies, mice were sacrificed by a lethal injection of sodium pentobarbital followed by exsanguination. After opening the thoracic cavity, trachea, lungs and heart were removed en bloc. Lungs were processed for bronchoalveolar lavage fluid (BALF) cell count as previously outlined [29], histological, biochemical or molecular biology studies. The right ventricular (RV) wall of the heart was dissected free and weighed along with the left ventricle wall plus septum (LV + S), and the resulting weights are reported as RV/LV + S ratio to provide an index of right ventricular hypertrophy. To determine the extent of pulmonary vascular remodeling, the degree of muscularization of intraacinar pulmonary vessels was determined. Lung sections (4 μm thickness) were stained with Masson’s trichrome and analysed using a morphometric system (Olympus BH2 Research Microscope, Olympus America Inc, Center Valley, PA, USA) with the software package Image ProPlus 5.0 (MediaCybernetics, Silver Spring, MD, USA). In each animal, 25–40 intraacinar arteries with an external diameter between 20 and 50 μm were analysed. The measurements made include internal area (IA), the external perimeter (EP) and the external area (EA), defined by the outer edge of the smooth muscle layer. The absolute wall area (WA) was calculated with the following formula: WA = EA−IA [30]. All measurements were made by the same observer. The pulmonary artery wall thickness was calculated by dividing the WA by the EP as previously outlined [30].

### Hydroxyproline assay

Hydroxyproline concentration was determined in mouse lung tissue using a commercially available Hydroxyproline Assay Kit (Sigma Aldrich; catalog no. MAK008), according to the manufacturer’s protocol. In brief, 10 mg of mice lung tissue were homogenized in 100 μL of deionized water and hydrolyzed by addition of 100 μl of concentrated hydrochloric acid (HCl, ~ 12 M), at 120 °C for 3 h. Hydrolyzed sample was mixed and centrifuged at 10,000 *g* for 3 min and, 20 μL of supernatant were transfered to a 96 well plate, which was placed in a 60 °C oven to dry samples. Next 100 µl Chloramine T / Oxidation buffer mixture was added per well and incubated for 5 min. Finally, 100 µl of the 4-(dimethylamino) benzaldehyde (DMAB) solution was added per well. The final assay volume (200 µl) was incubated for 90 min at 60 °C. The reaction of oxidized hydroxyproline with DMAB results in a product with a colorimetric absorbance at 560 nm that is proportional to the hydroxyproline content of the samples. Hydroxyproline (µg/ml) is obtained from a standard curve. Results were expressed as µg/ml hydroxyproline / 1 mg of lung tissue.

### Chemotaxis assay

To quantify the effect of IL-11 on chemotactic migration we used the commercial CytoSelect ™ 96-Well Cell Migration Assay kit, pores diameter, 8 μm (Cell Biolabs, San Diego, CA). Fibroblasts isolated from the lung parenchyma of Tie2-GFP mice were placed on these membranes with DMEM 0.1% SFB cell culture medium. The upper plate with the fibroblasts was coupled to a lower plate whose wells contained: the chemoattractant CXCL12 (recombinant mouse (rm)CXCL12, cat. n. 460-SD-010/CF) at a concentration of 100 ng/mL or rmIL-11 5 ng / ml or rmIL-11Rα 10 ng / ml or rmIL-11 + rmIL-11Rα. The plates were incubated for 6 h at 37 °C with 5% CO2 in humidified air. After this time, the upper plate was separated and attached to a new lower plate with a release buffer, so that those cells that had migrated through the polycarbonate membrane were detached. These detached cells were lysed and the CyQUANT®GR marker was added. Finally, fluorescence was quantified using a spectrophotometer (Infinite M200, Tecan) at 480 nm / 520 nm.

### Statistical analysis

Statistical analysis of results was carried out by non-parametric analysis. *P* < 0.05 was considered statistically significant. Data were displayed as medians and interquartile range values. When the comparisons concerned more than two groups, analysis of variance (Kruskal–Wallis test) was first performed. In the case of a global significant difference, between-group comparisons were assessed by the Dunn’s post-hoc test, which generalizes the Bonferroni adjustment procedure. When the comparisons concerned only 2 groups, between-group differences were analyzed by the Mann Whitney test. Correlation was analyzed by Spearman rho non-parametric analysis.

## Results

### IL-11 and IL-11Rα are increased in pulmonary arteries of patients with idiopathic pulmonary fibrosis and pulmonary hypertension

**Fig. 1:**
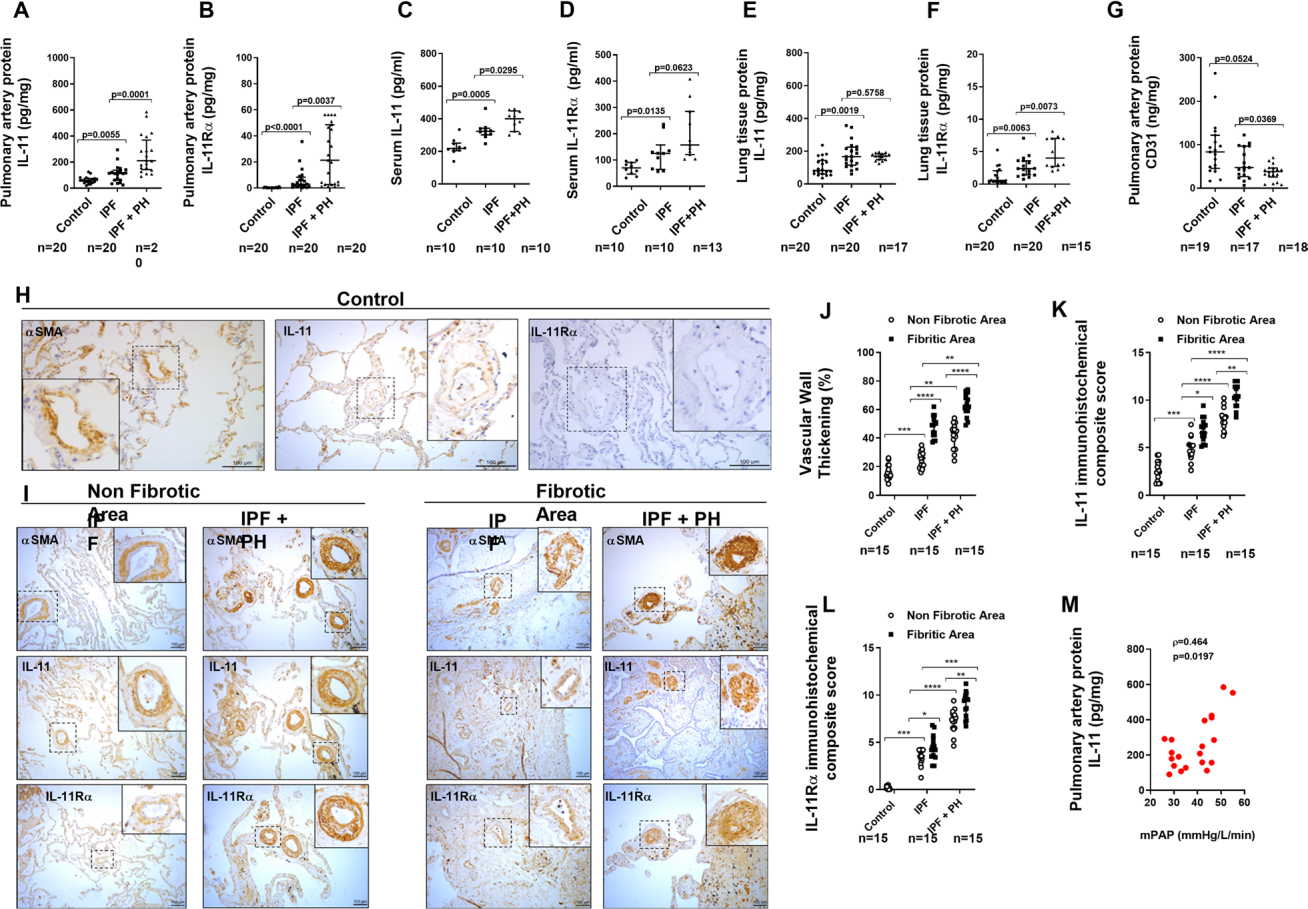
IL-11 and IL-11Rα are increased in whole lung homogenates, isolated pulmonary arteries and serum of patients with idiopathic pulmonary fibrosis (IPF) and pulmonary hypertension (PH) associated to IPF. The protein expression of IL-11 and IL-11Rα in **A**, **B** isolated pulmonary arteries (70–500 µm of internal diameter), **C**, **D** serum, and **E**, **F** lung tissue homogenates. Protein expression was measured using ELISA kits. **H** CD31 protein expression was measured in isolated pulmonary arteries as endothelial cells marker by ELISA. **H**, **I** Human lung tissue from control subjects, IPF and PH associated to IPF was immune-stained with IL-11, IL-11Rα and alpha smooth muscle actin (αSMA) antibodies. Representative images are showed from non-fibrotic lung areas and fibrotic areas. **J** Vascular wall thickening was quantified in a total of 20–30 pulmonary arteries per patient. **K**, **L** Immunohistochemical score quantification of IL-11 and IL-11Rα in a total of 20–30 pulmonary arteries per patient. Scale bar: 100 µm. Data are presented as scatter dot blot with median and interquartile range values. *P*-values are based on the Kruskal–Wallis test and Dunn’s post-hoc test for multiple comparison. **M** Spearman ρ correlation of IL-11 expression in isolated pulmonary arteries from PH + IPF and mean pulmonary artery pressure (mPAP). N indicates the number of patients in each graph

**Fig. 2:**
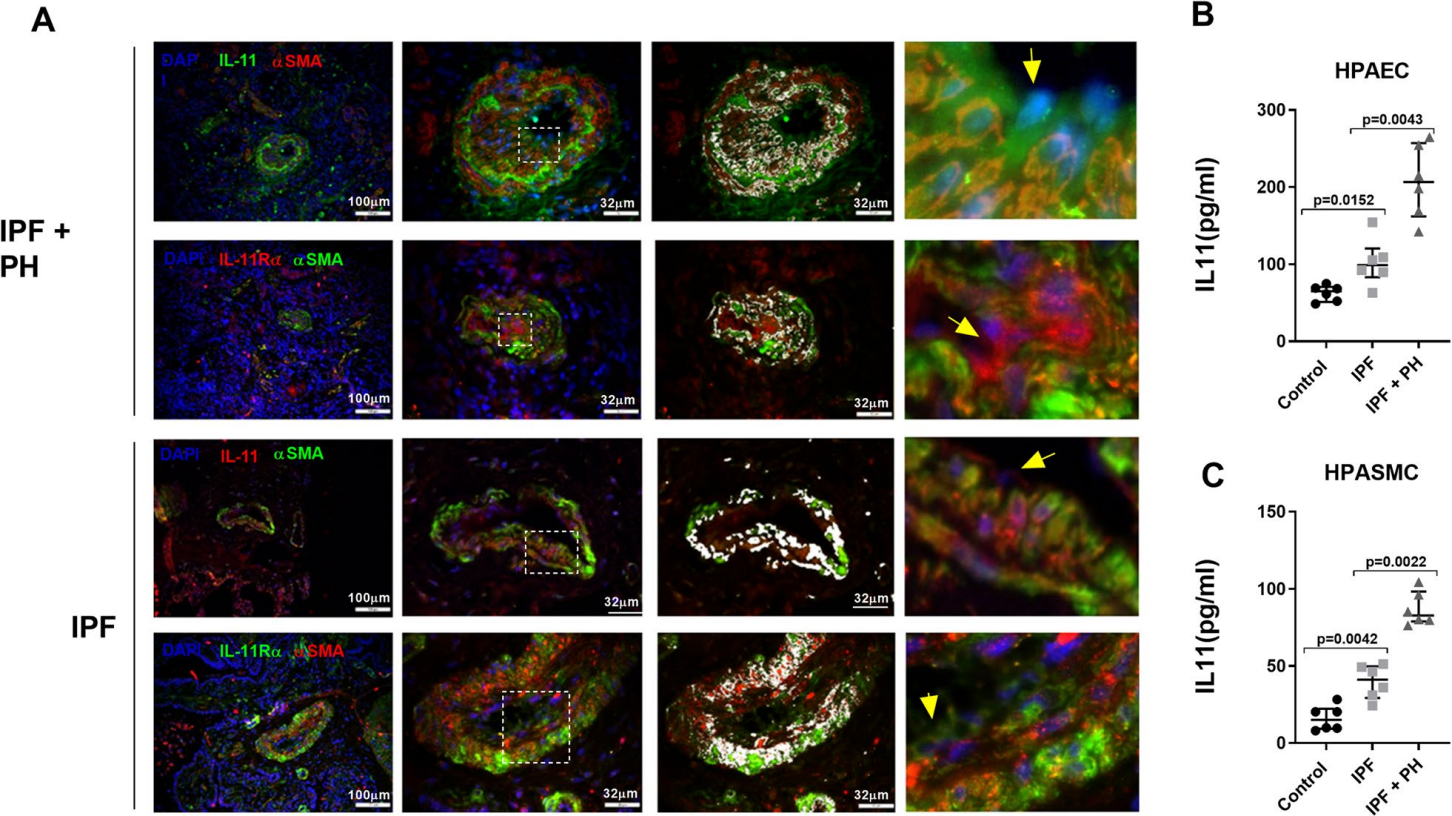
IL-11 and IL-11Rα are localized and secreted by human pulmonary artery endothelial cells (HPAEC) and smooth muscle cells (HPASMC). **A** Human lung tissue from control subjects, idiopathic pulmonary fibrosis (IPF) and pulmonary hypertension (PH) associated to IPF was immune-stained with IL-11, IL-11Rα and αSMA and with secondary fluorescence antibodies. Representative images are showed. White colour represents co-localization of both antibodies. Yellow arrows indicate endothelial cells. **B** HPAECs and **C** HPASMCs were isolated from pulmonary arteries of control subjects, IPF and PH associated to IPF patients and cultured until passage 1. Cell culture supernatants were collected to measure IL-11 by ELISA. Data are presented as scatter dot blot with median and interquartile range values of n = 6 patients in each group. *P*-values are based on the Kruskal–Wallis test and Dunn’s post-hoc test for multiple comparison

A total of 20 control subjects, 20 IPF and 20 IPF + PH patients were recruited. The levels of FVC%, DLco% and PaO_2_, were significantly lower in IPF + PH patients, whereas levels of mPAP were higher (Additional file 8: Table S4) confirming a more severe disease in IPF + PH patients. The IL-11 and IL-11Rα proteins were increased in isolated muscular pulmonary arteries (70–500 μm diameter) from IPF and IPF + PH (Fig. 1A; *P* < 0.01 *vs* control subjects), and significantly overexpressed in pulmonary arteries from IPF + PH patients (Fig. 1A and B; *P* = 0.01 vs IPF patients). Similarly, serum levels of IL-11 and soluble IL-11Rα were increased in IPF and IPF + PH patients compared with control subjects, and more elevated in IPF + PH than in IPF patients (Fig. 1C and D). In whole lung homogenates, the expression of IL-11 was similar in IPF and IPF + PH, whilst IL-11Rα was more elevated in IPF + PH than in IPF patients (Fig. 1E and F). In addition, the expression of the endothelial cell marker CD31 was downregulated in isolated pulmonary arteries from IPF + PH patients compared with IPF and control subjects (Fig. 1G). The analysis of histological samples of lung tissue showed that vascular wall thickening was elevated in distal pulmonary arteries of non-fibrotic areas from lungs of IPF- + PH patients compared with IPF and control subjects, but wall thickening was also present in IPF indicating an increase of pulmonary artery remodeling in absence of PH (Fig. 1H–J). In fibrotic areas, vascular wall thickening was higher than in non-fibrotic areas in both IPF and IPF + PH patients (Fig. 1H–J). The IL-11 and IL-11Rα immunohistochemical composite score was elevated in pulmonary arteries from IPF + PH, independently of the fibrotic or not fibrotic lung area analyzed, and significantly higher than in IPF pulmonary arteries (Fig. 1K and L). In addition the expression of IL-11 in pulmonary arteries of IPF + PH patients correlated with the mean pulmonary artery pressure (mPAP; Fig. 1M). Both IL-11 and IL-11Rα co-localized with pulmonary artery smooth muscle cells (Fig. 2, white color localization) and were also expressed in endothelial cells of pulmonary arteries (Fig. 2, yellow arrows) of IPF + PH and IPF patients. In addition, human pulmonary artery endothelial cells (HPAEC) and human pulmonary artery smooth muscle cells (HPASMC) isolated from IPF + PH showed the highest IL-11 release to the cell culture followed by cells from IPF patients and lower levels from control subjects (Fig. 2B and C).

### IL-11 induce in vivo pulmonary hypertension associated to pulmonary fibrosis with the participation of endothelial to mesenchymal transition process

**Fig. 3:**
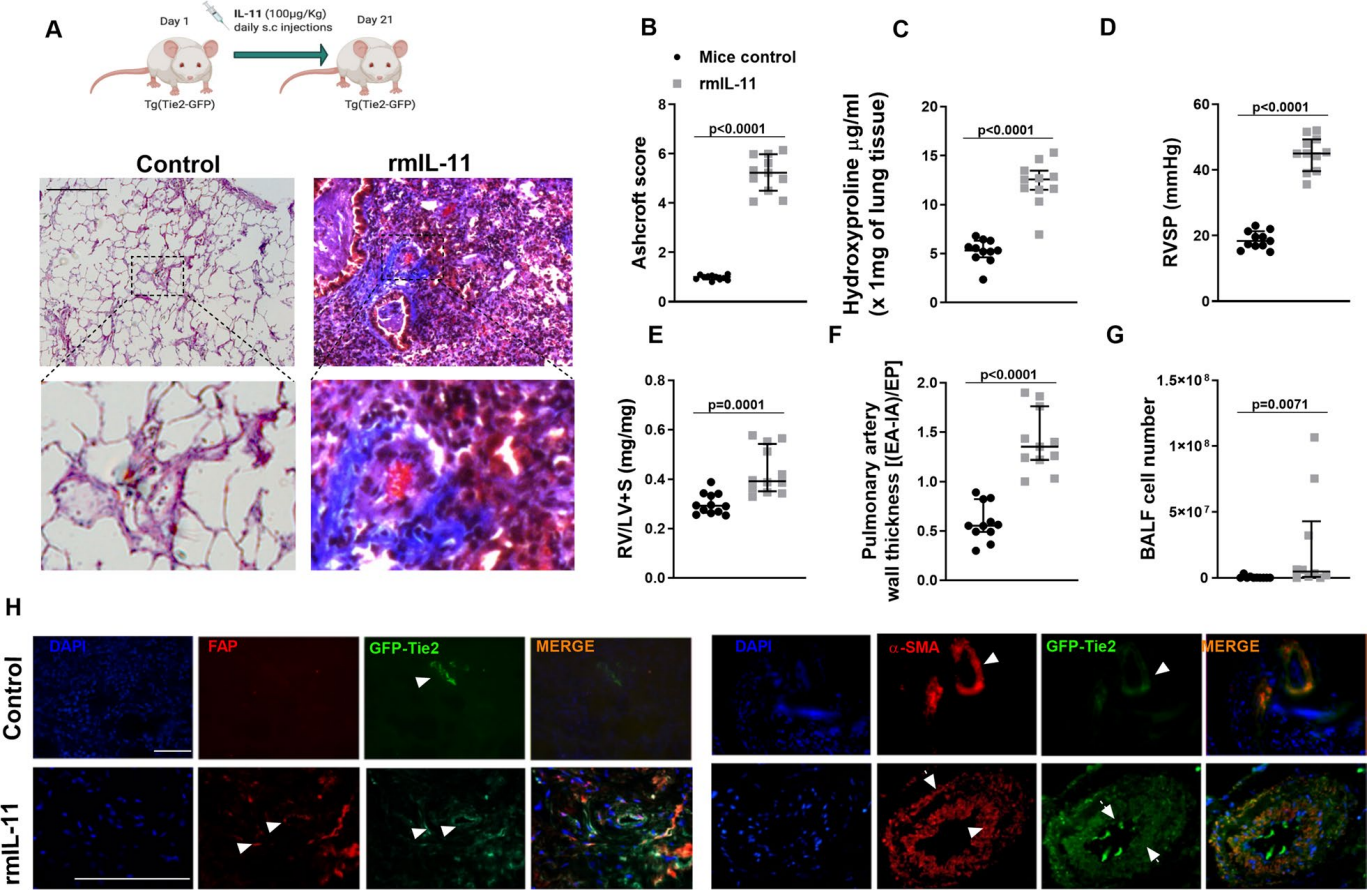
mrIL-11 induces lung fibrosis, pulmonary artery remodeling and hypertension. **A** A total of 100 µg/Kg/day of rmIL-11 (n = 11) or saline (n = 11) was subcutaneously administered during 21 days. At day 21 the following parameters were measured. **A** Masson’s trichrome histological images are showed. Scale bar: 100 µm. **B** Ashcroft score lung fibrotic index, **C** hydroxyproline amount in lung tissue, **D** right ventricular systolic pressure (RVSP) mmHg, **E** right ventricular (RV) hypertrophy measured by the ratio of RV/left ventricular (LV) + septo in mg/mg, **F** Pulmonary artery remodeling and **G** inflammatory cells in bronchoalveolar lavage fluid (BALF) were measured. **H** Co-immunofluorescence of FAP/Tie2-GFP and αSMA/Tie2-GFP. Scale bar: 100 µm. White arrows indicates co-localizations. Data are presented as scatter dot blot with median and interquartile range values. *P*-values are based on the Mann Whitney test

In this work, Tie2-green fluorescence protein (GFP) mice were used to genetically mark endothelial cells to follow the migration and contribution to pulmonary artery remodeling and lung fibrosis promotion. RmIL-11 (100 µg/kg/daily) was subcutaneously administered to Tie2-GFP mice during 21 days. RmIL-11 administration induced evident lung fibrosis and pulmonary artery remodeling as indicated the increase of Ashcroft score of lung histology (P < 0.05 vs control; Fig. 3A and B) and hydroxyproline lung concentration (Fig. 3C). The hemodynamic analysis was performed by right heart catheterization at day 21 of the animal procedure. Right ventricular systolic pressure (RVSP) was elevated in mice treated with rmIL-11 (46.1 ± 5.4 mmHg) compared with controls (19.25 ± 1.9 mmHg; *P* < 0.05; Fig. 3D). Right ventricular (RV) hypertrophy (RV/left ventricular (LV) + septum) and pulmonary vascular remodelling developed following rmIL-11 administration (Fig. 3E and F). The content of inflammatory cells in the bronchoalveolar lavage fluid was higher in rmIL-11 treated mice than in controls (Fig. 3G). Immunofluorescence analysis indicate that GFP-positive cells were localized in the endothelium of lung vessels of control mice, whereas in rmIL-11 treated mice, fibrotic FAP and α-SMA markers co-localized with GFP not only in endothelial cells but in neointimal, media and adventitia, with positive cells in parenchyma (Fig. 3H).

To further investigate the contribution of endothelial cells to lung fibrosis, parenchyma fibroblasts from control and rmIL-11 treated mice were isolated and cultured in vitro. Fibroblasts from rmIL-11 mice group showed higher expression of collagen type I, fibronectin, αSMA and IL-11Rα than controls (Additional file 1: Fig. S1A; *P* < 0.05) and possessed greater basal migratory capacity as well as following the stimulation with CXCL12, rmIL-11 or soluble rmIL-11Rα (Additional file 1: Fig. S1B). Immunoflorescence analysis showed that a proportion of parenchymal fibroblast isolated from rmIL-11 treated Tie2-GFP mice has endothelial origin as determined by co-expression of FAP/αSMA with GFP positive cells (Additional file 1: Fig. S1C). The expression of profibrotic markers and IL-11Rα were increased in whole lung homogenates of rmIL-11 mice group, while endothelial markers were downregulated (Additional file 2: Fig. S2A). The activation of the cell signal pathways p-ERK1/2, p-JAK2/p-STAT3, p-AKT and p-SMAD2/3 were increased in lung tissue of mice treated with rmIL-11 compared with control animals (Additional file 2: Fig. S2B).

**Fig. 4:**
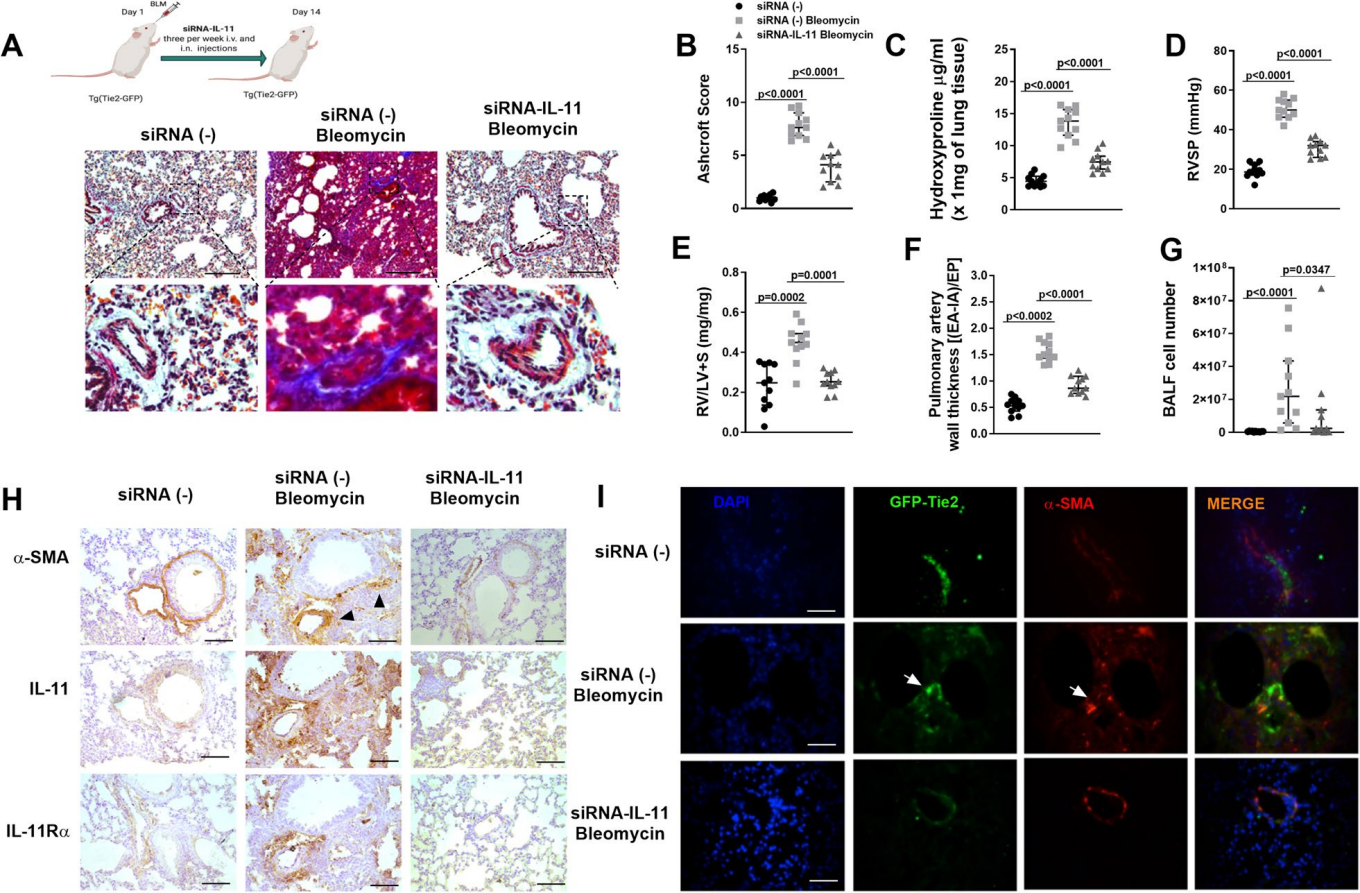
SiRNA-IL-11 transiently transfection attenuates bleomycin-induced lung fibrosis and pulmonary hypertension in transgenic Tie2-GFP mice. Wild-type (WT) siRNA(−) Tie2-GFP mice and IL-11-KO siRNA-IL-11 Tie2-GFP mice received a single intratracheal dose of bleomycin (1.5 U/kg) on day 1 (*n* = 11) during 14 days. siRNA-IL-11 was administered intravenously and intranasally three times a week from day 1 to day 14. At day 14 the following parameters were measured. **A** Masson’s trichrome histological images are showed. Scale bar: 100 µm. **B** Ashcroft score lung fibrotic index, **C** hydroxyproline amount in lung tissue **D** right ventricular systolic pressure (RVSP) mmHg, **E** right ventricular (RV) hypertrophy measured by the ratio of RV/left ventricular (LV) + septo in mg/mg, **F** pulmonary artery remodeling and **G** inflammatory cells in bronchoalveolar lavage fluid (BALF) were measured. **H** Immunohistochemical analysis of αSMA, IL-11 and IL-11Rα. Scale bar: 50 µm. Black arrows show pulmonary arteries. **I** Co-immunofluorescence of αSMA/Tie2-GFP. Scale bar: 25 µm. White arrows indicates co-localizations. Data are presented as scatter dot blot with median and interquartile range values. *P*-values are based on the Kruskal–Wallis test and Dunn’s post-hoc test for multiple comparison

Intratracheal bleomycin mice model produce lung fibrosis and pulmonary hypertension reproducing pathological conditions of human IPF associated to PH [26]. To study the role of IL-11 on PH associated to lung fibrosis, mice were transfected with siRNA-IL-11 to generate transient IL-11-KO mice (intranasal and intravenous injections three times/week). Bleomycin induced a robust lung fibrotic response as it indicated the increase of ashcroft score of lung histology (*P* < 0.05 vs siRNA(−) control; Fig. 4A and B) and hydroxyproline lung concentration (Fig. 4C) accompanied with evident pulmonary artery remodelling. SiRNA-IL-11 alleviated histologically observed multifocal fibrotic lesions, with a diminished ashcroft fibrosis score and hydroxyproline lung concentration (*P* < 0.05 vs bleomycin group; Fig. 4A–C), and pulmonary artery remodelling. Right heart catheterization showed an increase of RVSP in the group treated with bleomycin (50.2 ± 5.6 mmHg; Fig. 4D) compared with siRNA (−) controls (19.17 ± 1.8 mmHg; *P* < 0.05; Fig. 4D), and was reduced by the treatment with siRNA-IL-11 (31.6 ± 1.2 mmHg; P < 0.05 vs Bleomycin siRNA(−) group). In addition, siRNA-IL-11 reduced the bleomycin-induced right ventricular (RV) hypertrophy (RV/left ventricular (LV) + septum) and pulmonary vascular remodeling (Fig. 4E, F). Bleomycin increased the expression of IL-11 and IL-11Rα that was mainly located in remodeled pulmonary arteries, showing positivity on pulmonary artery endothelial and smooth muscle cell as well as in other αSMA positive cells in lung parenchyma (Fig. 4H). Tie2-GFP-positive cells were localized in the endothelium of lung vessels of control mice, whereas in bleomycin siRNA(−) mice, α-SMA/GFP co-localized not only in endothelial cells but also in lung parenchyma (Fig. 4I). The treatment of mice with siRNA-IL-11 reduced parenchymal GFP positive cells (Fig. 4I).

Mouse fibroblasts isolated from lung parenchyma of bleomycin siRNA(−)/Tie2-GFP mice group showed an increase of IL-11, IL-11Rα, fibronectin and collagen type I that was reduced in fibroblasts from siRNA-IL-11 bleomycin treated mice (Additional file 3: Fig. S3A). In addition, lung fibroblasts from the bleomycin siRNA(−) group showed an increased basal migration as well as following rmIL-11 and soluble rmIL-11Rα stimulation (Additional file 3: Fig. S3B), that was reduced in fibroblasts from siRNA-IL-11 bleomycin treated mice.

In contrast to fibroblasts isolated from siRNA(−), part of the isolated lung fibroblasts from siRNA(−)-bleomycin mice were positive for FAP/αSMA and GFP supporting an endothelial origin. The number of FAP/αSMA and GFP positive fibroblasts was suppressed in siRNA-IL-11-bleomycin treated mice (Additional file 3: Fig. S3C).

In whole lung homogenates, siRNA-IL-11 inhibited the bleomycin-induced increase of profibrotic mRNA transcripts and rescued from the loss of endothelial cell markers (Additional file 4: Fig. S4A). In a similar way, siRNA-IL-11 transfection inhibited the effects of bleomycin on the activation of cell signal pathways p-ERK1/2, p-JAK2/p-STAT3, p-AKT and p-SMAD2/3 (Additional file 4: Fig. S4B).

### IL-11 and IL-11Rα induce endothelial to mesenchymal transition and pulmonary artery smooth muscle-myofibroblast like transformations

**Fig. 5:**
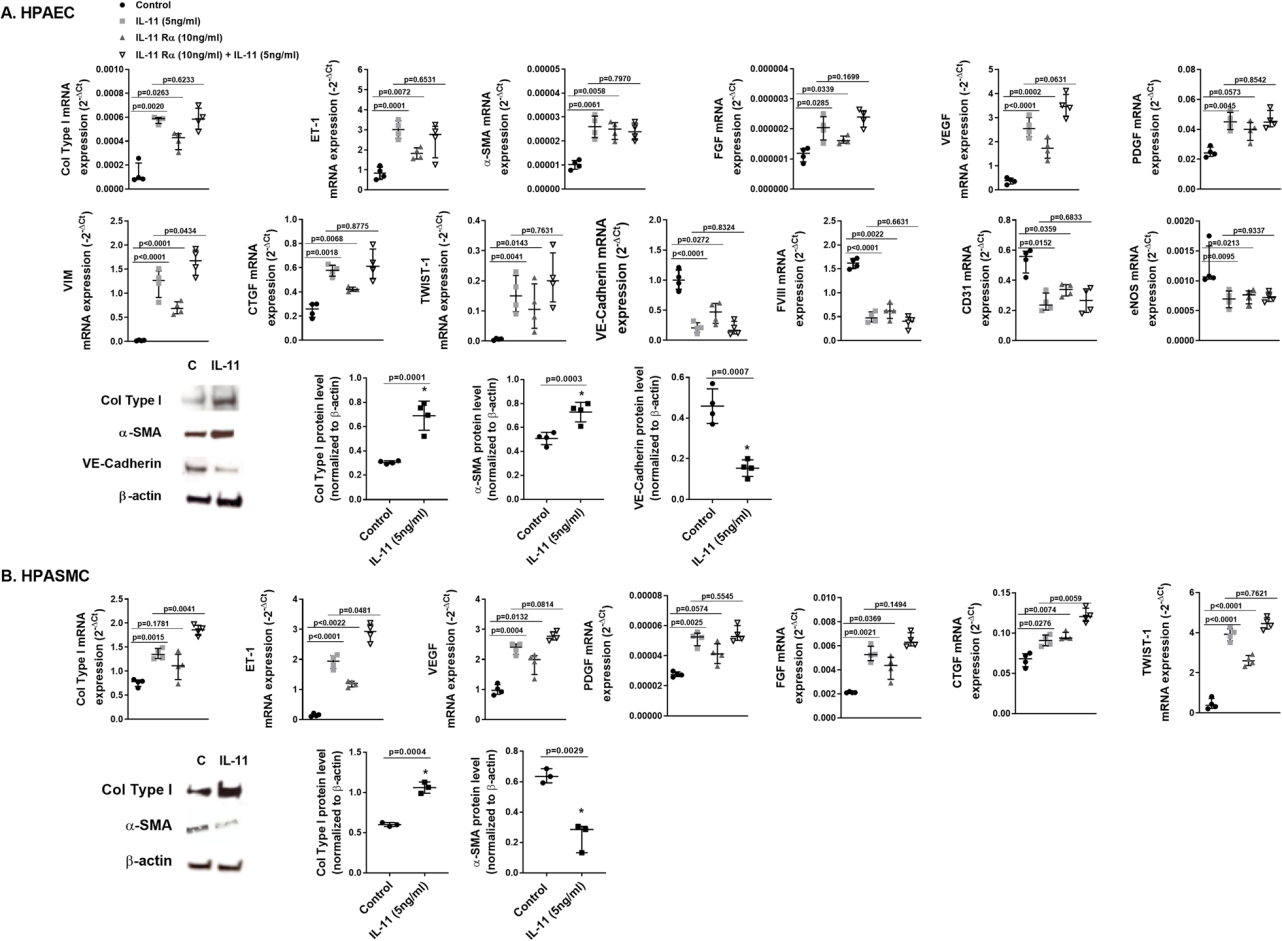
IL-11 and soluble IL-11Rα induce human pulmonary artery endothelial cell (HPAEC) to mesenchymal transition (EnMT) and human pulmonary artery smooth muscle cell (HPASMC) to myofibroblast-like transition. **A** HPAEC and **B** HPASMC were isolated from control donor subjects and stimulated with rhIL-11 5 ng/ml, rhIL-11Rα 10 ng/ml or their combination during 48 h replacing culture medium and stimulus each 24 h. Experiments were done between passages 2–3. Gene mRNA transcripts of different genes measured by quantitative PCR (qPCR) as 2^−ΔCt^. Protein expression levels were analysed by western blotting. Data are shown as the ratio compared to β-actin for protein. Data are presented as scatter dot blot with median and interquartile range values (for primary cells, *n* = 4 control subjects performed in triplicate). *P*-values are based on the Mann Whitney test (two groups) or the Kruskal–Wallis test and Dunn’s post-hoc test for multiple comparison

The EnMT process contributes to the pulmonary artery remodeling and endothelial dysfunction in patients with pulmonary hypertension [31, 32]. To explore direct effect of IL-11 and soluble IL-11Rα on EnMT, and HPASMC-myofibroblast like acquired phenotype, we first incubated primary culture cells from control subjects with human recombinant IL-11 (hrIL-11) (5 ng/ml, 48 h), soluble hrIL-11Rα (10 ng/ml, 48 h) or their combination. In HPAECs, HrIL-11 and hrIL-11Rα induced TWIST-1, cytoskeleton αSMA and vimentin overexpression, extracellular collagen type I and FGF, PDGF, CTGF and VEGF growth factor transcription increase, accompanied with an increase of vasoactive endothelin 1(ET-1) and reduction of eNOS vasodilator and endothelial markers such as CD31, VE-cadherin and FVIII (Fig. 5A), supporting an EnMT process. Transcription modifications were accompanied with protein modifications (Fig. 5B). In HPASMC, rhIL-11 and soluble hrIL-11Rα increased the expression of TWIST-1, collagen type I and the growth factors FGF, PDGF, CTGF, VEGF and vasoactive ET-1 (Fig. 5C). Contractile protein expression such has αSMA was decreased while extracellular matrix protein collagen type I was overexpressed (Fig. 5C, D). The combination of rhIL-11 and rhIL-11Rα appears to have additive effects but not for all markers of EnMT and HPASMC to myofibroblast-like transition.

**Fig. 6:**
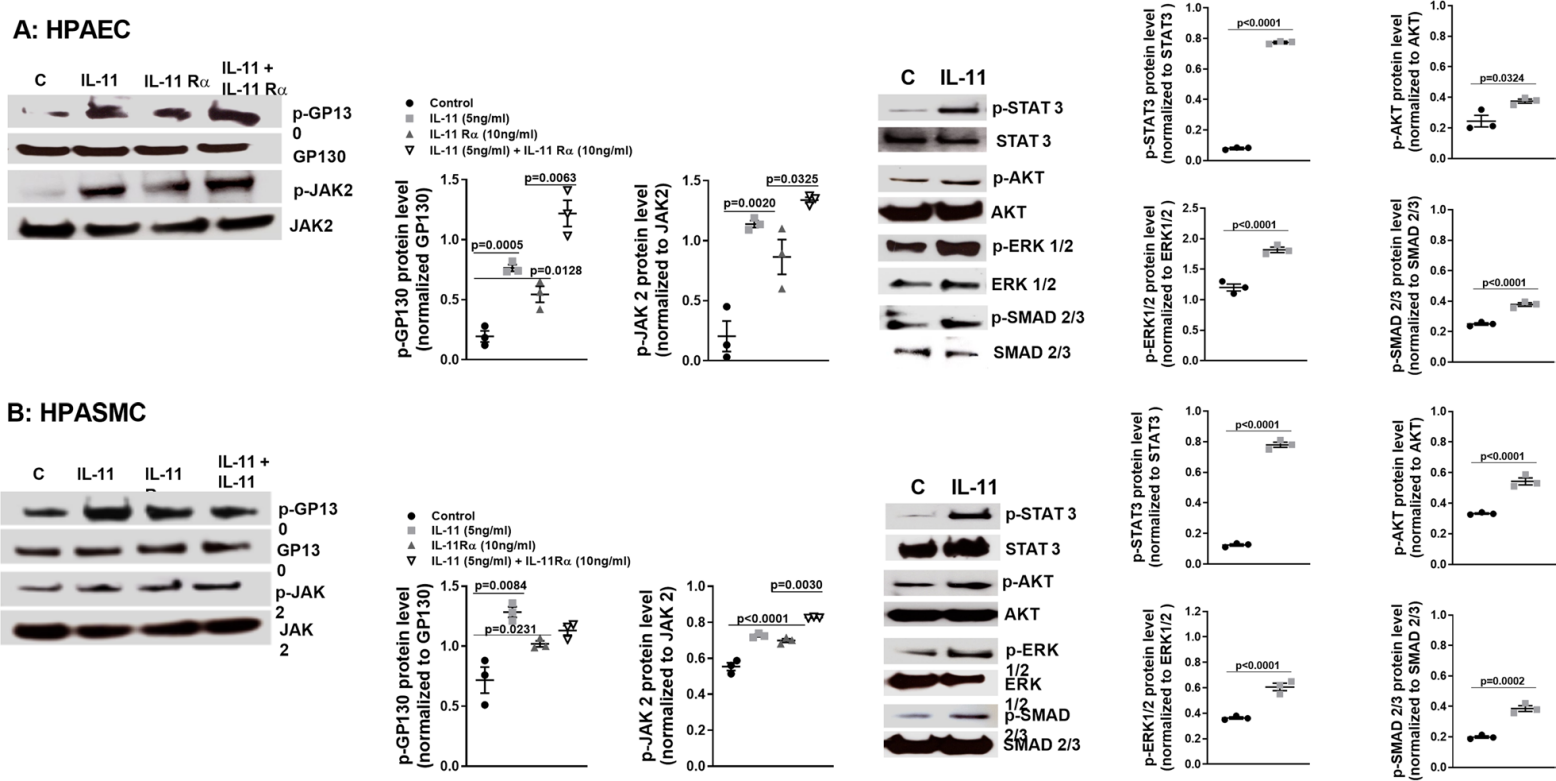
rhIL-11 and soluble rhIL-11Rα activates intracellular signal. **A** Human pulmonary artery endothelial cells (HPAEC) and **B** human pulmonary artery smooth muscle cells (HPASMC) were isolated from control donor subjects and stimulated with rhIL-11 5 ng/ml, rhIL-11Rα 10 ng/ml or its combination during 30 min. Experiments were done between passages 2–3. Protein expression levels were analysed by western blotting. Data are shown as the ratio compared to β-actin or non-phosphorylated protein as indicate. Representative blots are sowed. Data are presented as scatter dot blot with median and interquartile range values (for primary cells, *n* = 3 control subjects performed in triplicate). *P*-values are based on the Mann Whitney test (two groups) or the Kruskal–Wallis test and Dunn’s post-hoc test for multiple comparison

**Fig. 7:**
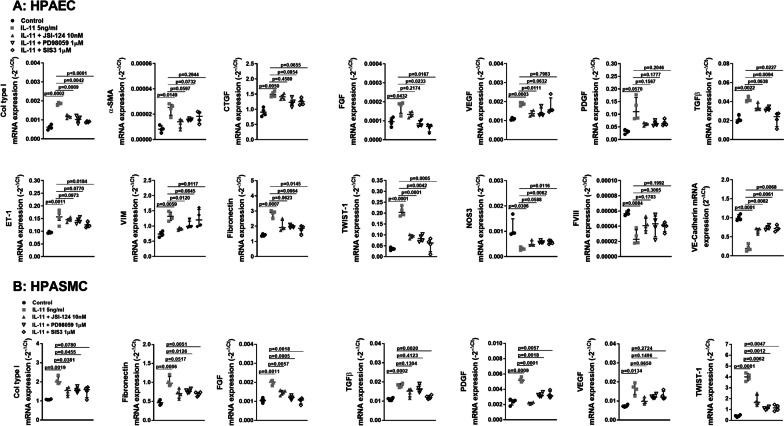
rhIL-11 mediates human pulmonary artery endothelial cell (HPAEC) to mesenchymal transition (EnMT) through the canonical JAK/STAT3 and non-canonical ERK/SMAD3 pathways. **A** HPAECs and **B** HPASMC were isolated from control donor subjects, incubated with a JAK/STAT3 inhibitor JSI-124, ERK inhibitor PD98059 or SMAD3 inhibitor SIS3 during 30 min, followed by the stimulation of cells with rhIL-11 5 ng/ml during 48 h. Experiments were done between passages 2–3. mRNA transcripts of different genes were measured by quantitative PCR (qPCR) as 2^−ΔCt^. Data are presented as scatter dot blot with median and interquartile range values (for primary cells, *n* = 4 control subjects performed in triplicate). *P*-values are based on the Kruskal–Wallis test and Dunn’s post-hoc test for multiple comparison

Previous reports have shown that IL-11 mediates its tissue fibrotic effects through the non-canonical ERK pathway [13, 14]. In this work we detected an activation of canonical GP130 and JAK2/STAT3 phosphorylations as well as non-canonical ERK1/2, AKT and SMAD2/3 phosphorylations after a short time (30 min) period of rhIL-11 stimulation in both HPAECs and HPASMCs (Fig. 6A, B). Soluble rhIL-11Rα showed similar effects than rhIL-11 phosphorylating GP130 and JAK2, and the combination of rhIL-11Rα and rhIL-11 produced additive effects activating GP130 and JAK2, which confirm a biological role for soluble IL-11Rα. To analyze which cellular signal promotes the induction of EnMT and HPASMC-myofibroblast-like cell transformation we incubated HPAECs and HPASMCs with an inhibitor of ERK (PD98059), JAK2/STAT3 (JSI124) or SMAD3 (SIS3). The three inhibitors attenuated the IL-11-induced EnMT and HPASMC-myofibroblast-like cell transformation with similar effect, suggesting the participation of canonical and non-canonical pathways (Fig. 7A, B).

### IL-11 and IL-11Rα modulates endothelial and smooth muscle cell proliferation and senescence

**Fig. 8:**
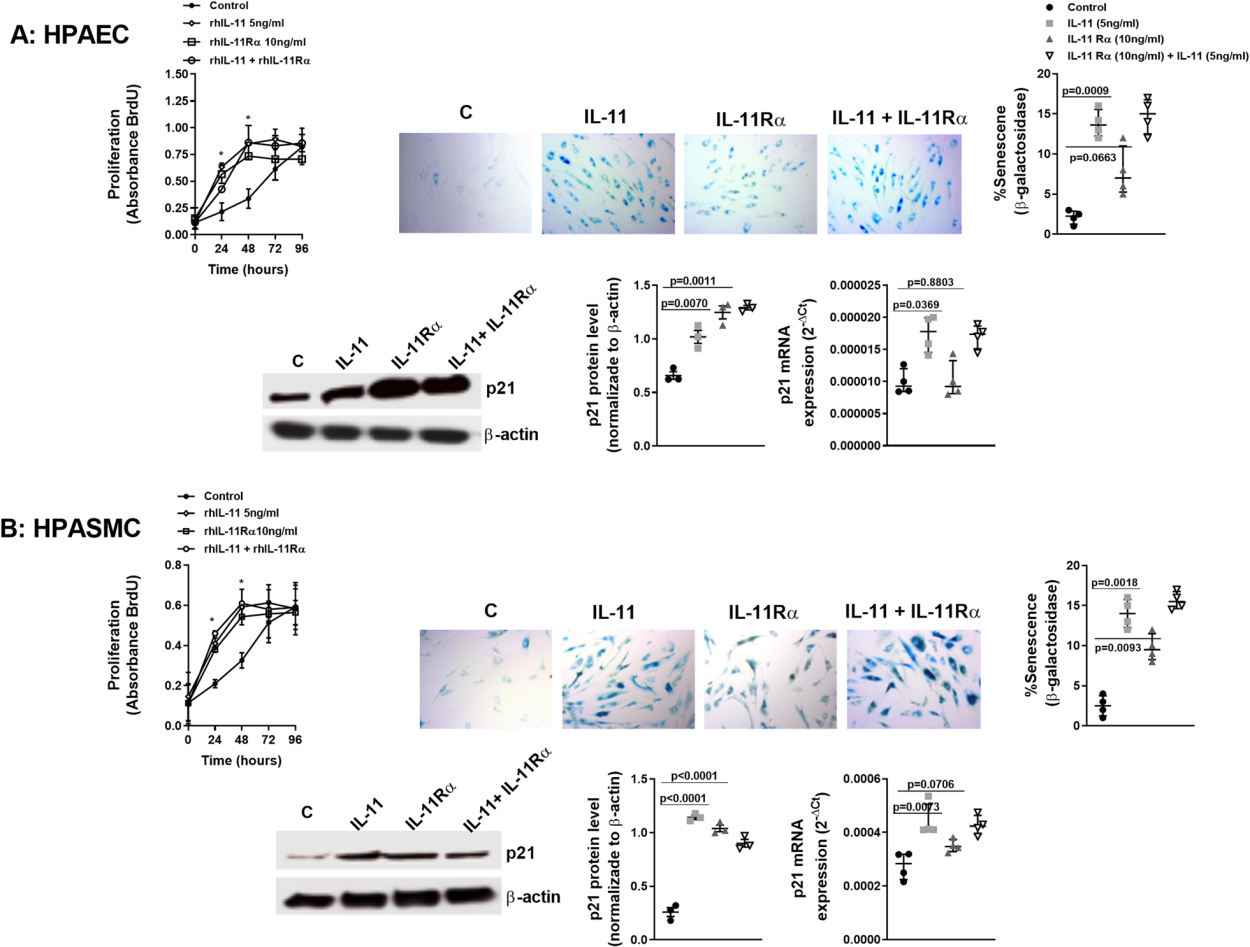
rhIL-11 and soluble rhIL-11Rα promotes time-dependent proliferation and senescence in human pulmonary artery endothelial cells (HPAEC) and smooth muscle cells (HPASMC). **A** HPAECs or **B** HPASMCs were isolated from control donor subjects and stimulated with rhIL-11 5 ng/ml, rhIL-11Rα 10 ng/ml or its combination at indicated times. Experiments were done between passages 2–3. Cell proliferation was measured by the BrDU kit at 24 h, 48 h, 72 h and 96 h. Cell senescence was measured after 72 h of cell stimulation using β-galactosidase histology and P21 expression. Results were expressed as % senescence (β-galactosidase blue positive cells) relative to the total number of cells in each field. P21 expression was measured by quantitative PCR (qPCR) as 2^−ΔCt^ and western blot. Data are presented as scatter dot blot with median and interquartile range values (for primary cells, *n* = 3–4 control subjects performed in triplicate). *P*-values are based on the Kruskal–Wallis test and Dunn’s post-hoc test for multiple comparison

Primary pathologic features of vascular remodeling in PH are hyperproliferation and apoptosis resistance of vascular cells, accompanied by an extensive profile of proinflammatory cytokines [33]. In addition to the proliferative phenotype, senescence-associated secretory phenotype (SASP) is also present in pulmonary arteries of lung chronic diseases such as IPF, contributing to pulmonary artery remodeling [34, 35]. In this work rhIL-11 induced a time-dependent increase of HPAECs and HPASMCs proliferation that reach a maximum increase at 48 h of stimulation (Fig. 8A and B; *P* < 0.05 vs control), showing proliferation arrest at 72 h and 96 h after stimulation. Similar results were observed when cells were stimulated with rhIL-11Rα or its combination with rhIL-11. Next we analyzed whether the cell growth arrest at 72 h could be overlapped with a senescence phenotype. RhIL-11 and rhIL-11Rα induced an increase of β-galactosidase positive cells with an increment of the senesce marker P21 in both HPAECs and HPASMCs (Fig. 8A, B). The analysis of isolated pulmonary arteries of different patients showed an increased expression of P21 and P16 senescence markers in IPF, and overexpressed in IPF + PH (p < 0.01 vs IPF; Additional file 5: Fig. S5A, B). In addition, P21 and P16 gene expression correlated with IL-11 expression in IPF + PH pulmonary arteries (Additional file 5: Fig. S5C, D). The correlation of P21 and IL-11 expression was accompanied by some degree of pulmonary artery co-localization in pulmonary arteries from IPF + PH patients (Additional file 5: Fig. S5E). In mice, rmIL-11 treatment increased the expression of both, P21 and IL-11 that were partially co-localized (Additional file 2: Fig. S2B and Additional file 6: Fig. S6A). Similar results were observed in the pulmonary arteries from bleomycin treated mice, in which siRNA-IL-11 decreased the P21 expression in pulmonary arteries (Additional file 4: Fig. S4B and Additional file 6: Fig. S6B, Additional file 7, 9).

## Discussion

Using control, IPF and IPF with PH lungs from human and PH animal models, we showed for the first time that IL-11/IL-11Rα may be implicated in the development of PH associated to IPF chronic lung disease.

The biological mechanisms involved in development of PH are multifactorial and complex and not yet fully understood. Recent studies have revealed IL-11 as a key fibrotic mediator in different organs including lung [13, 14], however, there is no evidence on the role of IL-11 on pulmonary arteries and PH. In this work, isolated pulmonary arteries from IPF associated to PH showed the highest expression of IL-11 and IL-11Rα and correlated with mPAP. The overexpression was observed in pulmonary arteries from non-fibrotic lung areas of IPF + PH patients, but increased in pulmonary arteries with a more degree of vascular wall thickening such as those located in fibrotic areas. In addition, IL-11 and IL-11Rα were localized in different layers of pulmonary artery suggesting a contribution on endothelial and smooth muscle cells. Of interest, we detected elevated levels of IL-11 and soluble IL-11Rα in serum of IPF and IPF associated to PH. Part of the levels detected in serum can be released by HPAEC and HPASMCs as we measured in primary culture cells of different patients, but other cell types could also contribute [36]. As occurs with other interleukins of the IL-6 family [37], soluble IL-11Rα can be detected in serum [38], but their function is completely unknown. Recent studies suggest that IL-11Rα can be cleaved by ADAM10 from the cell surface to produce a soluble form of IL-11Rα that is present in the serum, allowing for trans-signaling of IL-11 on cell types that do not normally express the receptor [38]. Supporting this data, ADAM10 is elevated in IPF [39] and participates in PAH [40], which could explain the elevated levels of soluble IL-11Rα we detected in this work, although we did not formally explored this hypothesis. Therefore, two different mechanisms, cluster signaling and trans-signaling, may allow IL-11 to act on a broad range of cells that express, or not, membrane bound IL-11Rα.

The main histologic alterations in PH are neointimal vascular remodeling, hypertrophied media, and inflammation and fibrosis of the adventitia. Endothelial and smooth muscle cells participate in all these processes, although the role of these cell types in the ILD/PAH pathobiology is incompletely understood. Recent evidence indicate that EnMT participates in PAH [32] and also in lung fibrosis [7]. Furthermore, we previously observed markers of EnMT in pulmonary arteries of IPF patients with PH contributing to neointimal formation [17, 26]. In a similar way, in PAH, dynamic and maladapted pulmonary vascular remodeling is accompanied by the acquisition and maintenance of HPASMCs proliferation, migration and secretion of extracellular matrix, growth factors and inflammatory molecules, losing the physiologic quiescent/ contractile phenotype represented by the expression of αSMA and smooth muscle-myosin heavy chain expression. These cellular processes promotes media hypertrophy and migration into the tunica intima assuming a myofibroblast-like (also called “synthetic”) phenotype which is an adaptive response but results in vessel wall thickening [35, 41]. Indeed, each individual HPASMC can reversibly go from a quiescent to a contractile or synthetic phenotype at any time and therefore acquire or lose proliferative, migratory and secretory extracellular matrix potential [42].

Previous reports showed that IL-11 promotes lung fibroblast to myofibroblast transition promoting fibrosis of the lung parenchyma [14]. In this work we assumed the hypothesis that IL-11 mediates pulmonary artery remodeling and hypertension promoting EnMT and HPASMC-myofibroblast-like transition that resembles to previously observed in lung fibroblasts. Since cells from IPF or iPAH can present some degree of phenotypic alterations, in this work we used HPAEC and HPASMC from control subjects to explore whether IL-11 or IL-11Rα are able to induce cell transformations. The use of healthy cells instead of cell from IPF/PH patients may represent a limitation of the study. Here, prolonged stimulation with IL-11 or its soluble receptor IL-11Rα induced transcript and protein alterations in HPAEC showing an increase of contractile and cytoskeleton proteins αSMA and vimentin, elevation of growth factors and vasoconstrictor protein ET-1, and decrease of endothelial cell markers compatible with the EnMT and endothelial dysfunction processes. Similar findings were observed in HPASMCs, where IL-11 or its soluble receptor IL-11Rα promoted a myofibroblast-like phenotype characterized by the increase of extracellular matrix expression, production of growth factors and ET-1, accompanied by a loss of αSMA. These results were similar to that observed using TGFβ as stimulus [26]. Supporting our findings, recent evidence indicate that IL-11 causes aortic vascular smooth muscle cell (VSMC) phenotypic switching [43]. IL-11 overexpression develops aortic remodelling increasing matrix and inflammatory genes expression and anti-IL11 antibodies reduced aortic remodeling in different mice models of arterial pressure loading [43].

In this work we observed that both, IL-11 and soluble IL-11Rα activates p-GP130 and p-JAK2 and that IL-11 phosphorylates STAT3, AKT, ERK1/2 and SMAD2/3. Assuming that the stimulations were implemented in a short period of time (30 min) we cannot confirm which phosphorylation occurs first. Using inhibitors of canonical JAK2/STAT3 (JSI-124) and non-canonical ERK (PD98059), or SMAD3 (SIS3) pathways, we observed that both canonical and non-canonical pathways are implicated in IL-11 and IL-11Rα-induced EnMT and HPASMC-myofibroblast-like transition. Previous reports have shown that IL-11 can activate canonical JAK2/STAT3 as well as non-canonical ERK1/2 and PI3K/AKT/mTOR pathways [44–46], all of them implicated in cell proliferation and pulmonary artery remodeling in PH [47–50]. Furthermore, it has been shown that JAK2 phosphorylation can trans-activate SMAD2/3 signalling [26, 51] which may explain how IL-11 and IL-11Rα activates SMAD2/3 phosphorylation.

In addition to cell transformations observed in this work, IL-11 and soluble IL-11Rα induced HPAEC and HPASMC proliferation that was progressive during 48 h, at which point proliferation stopped and both cell types showed an increase of senesce represented by the strong expression of β-galactosidase and P21. In addition, P21 and P16 senescence markers were overexpressed and correlated with IL-11 expression in pulmonary arteries from IPF + PH patients. PH associated to chronic lung diseases such as IPF and COPD are associated with an elevated levels of senescent cells [52] mixed with proliferative phenotypes in pulmonary arteries. Senescence of pulmonary artery cells has the inability to further proliferate, acquiring an inflammatory phenotype (labelled senescence-associated secretory phenotype or SASP), largely characterized by overexpression of pro-inflammatory cytokines and growth factors such as FGF and PDGF. SASP seemed to stimulate hypertrophy, hyperplasia, and migration of non-senescent smooth muscle cells in culture [52], all of which could contribute to vascular remodeling in PH [35].

To further characterize the role of IL-11 on PH, we used a Tie2-GFP mice stimulated with rmIL-11 during 21 days. In this model we observed an increase of pulmonary fibrosis accompanied with elevated RVSP, pulmonary artery remodeling and right ventricle hypertrophy. Interestingly, endothelial Tie2-GFP positive cells were found in the hypertrophied media and neointimal as well as in lung fibrotic parenchyma as represented by myofibroblast-like cells co-expressing Tie2-GFP/αSMA/FAP markers that showed an enhanced migratory phenotype. These results indicate an active role of EnMT in the process of pulmonary artery remodeling induced by IL-11. Similar results were observed when PH associated with PF was induced by intratracheal bleomycin administration. In this model, Tie2-GFP cells form endothelial origin participated in pulmonary artery remodeling and lung fibrosis as previously outlined [8]. Remodeled pulmonary arteries showed an increased expression of IL-11 and IL-11Rα as observed in human disease. Transiently transfection of siRNA-IL-11 reduced pulmonary artery remodeling and pulmonary hypertension as well as the number of positive Tie2-GFP cells in lung parenchyma which confirm the role of IL-11 on in vivo EnMT.

We have shown evidences on the role of IL-11 and IL-11Rα on PH associated to IPF. However, results from donor control subjects included in this study showed cardiovascular disorders as the cause of death, including cerebrovascular/stroke or acute myocardial infarction between others (see Additional file 8: Table S1) that could modify the vascular expression of IL-11/IL-11Rα, thus representing a limitation of this study. In addition, all samples used in this study were from lung end-stage stable disease as described in methods which have their advantages (uniformity of samples) and disadvantages (lung end-stage disease that not represents different stages and progression of disease). Furthermore, the main results provided in this work are provided from animal models with their limitations and cannot fully translated to the human IPF/PH disease.

## Conclusions

Current therapies for PAH are focused on vasodilators that are not useful to treat PH associated to IPF. Therefore new pulmonary artery anti-remodelling agents could be of potential benefit to ameliorate the progression of disease. This work introduce IL-11 and IL-11Rα as a drivers of pulmonary hypertension potentially druggable to reduce pulmonary artery remodeling.

## Supplementary Information

**Additional file 1: Figure S1**. mrIL-11 induces endothelial to mesenchymal transition (EnMT) *in vivo*, promoting parenchymal myofibroblast-like cells of endothelial origin. A total of 100µg/Kg/day of rmIL-11 (n=11) or saline (n=11) was subcutaneously administered during 21 days. At day 21 parenchymal lug fibroblast were isolated and cultured at passage 1. (A) The expression of extracellular matrix proteins, αSMA and IL-11Rα was measured by quantitative PCR (qPCR) as 2^−ΔCt^. (B) Isolated lung fibroblasts from sham control or mrIL-11 treated animals studied to analyse the migratory capacity following basal, rmCXCL12, rmIL-11, rmIL-11Rα or its combination. (C) Co-immunofluorescence analysis of FAP/tie2-GFP and αSMA/ tie2-GFP of isolated parenchymal fibroblasts. Scale bar: 10µm. Data are presented as scatter dot blot with median and interquartile range values. *P*-values are based on the (A) Mann Whitney or (B) Kruskal-Wallis test followed by Dunn’s post-hoc test for multiple comparison.

**Additional file 2: Figure S2. **mrIL-11 induces lung tissue remodeling increasing profibrotic markers and reducing endothelial cell markers. A total of 100µg/Kg/day of rmIL-11 (n=11) or saline (n=11) was subcutaneously administered during 21 days. At day 21 lung homogenates were processed to (A) measure profibrotic and endothelial cell markers presented by heat map representation of mRNA transcripts of different genes and measured by quantitative PCR (qPCR) as 2^−ΔCt^. *P < 0.05 *vs* sham controls (B) Intracellular signalling markers, collagen type I and senescence P21 marker protein expression by western blotting. Data are shown as the ratio compared to β-actin or non-phosphorylated protein as indicate. Data are presented as scatter dot blot with median and interquartile range values. *P*-values are based on the Mann Whitney test.

**Additional file 3:  Figure S3.** SiRNA-IL-11 transiently transfection reduces bleomycin-induced endothelial to mesenchymal transition (EnMT) *in vivo*, suppressing parenchymal myofibroblast-like cells of endothelial origin. Wild-type (WT) siRNA(-) tie2-GFP mice and IL-11-KO siRNA-IL-11 tie2-GFP mice received a single intratracheal dose of bleomycin (1.5 U/kg) on day 1 (*n *= 11) during 14 days. At day 14 parenchymal lug fibroblast were isolated and cultured at passage 1. (A) The expression of extracellular matrix proteins, IL-11 and IL-11Rα was measured by quantitative PCR (qPCR) as 2^−ΔCt^. (B) Isolated lung fibroblasts from sham siRNA(-) control, siRNA(-) bleomycin and siRNA-IL-11 bleomycin treated animals were studied to analyse the migratory capacity following the stimulation with basal medium, rmCXCL12, rmIL-11, rmIL-11Rα or its combination. (C) Co-immunofluorescence analysis of FAP/tie2-GFP and αSMA/ tie2-GFP of isolated parenchymal fibroblasts. Scale bar: 10µm. Data are presented as scatter dot blot with median and interquartile range values. *P*-values are based on the Kruskal-Wallis test followed by Dunn’s post-hoc test for multiple comparison.

**Additional file 4: Figure S4**. SiRNA-IL-11 transiently transfection attenuates bleomycin-induced lung tissue remodeling reducing profibrotic markers and increasing endothelial cell markers. Wild-type (WT) siRNA(-) tie2-GFP mice and IL-11-KO siRNA-IL-11 tie2-GFP mice received a single intratracheal dose of bleomycin (1.5 U/kg) on day 1 (*n *= 11) during 14 days. At day 14 lung homogenates were processed to (A) measure profibrotic and endothelial cell markers presented by heat map representation of mRNA transcripts of different genes and measured by quantitative PCR (qPCR) as 2^−ΔCt^. *P < 0.05 *vs* sham siRNA(-) controls; #*P* <0.05 vs siRNA(-) bleomycin mice group. (B) Intracellular signalling markers, collagen type I and senescence P21 marker protein expression by western blotting. Data are shown as the ratio compared to β-actin or non-phosphorylated protein as indicate. Representative blots are sowed. Data are presented as scatter dot blot with median and interquartile range values. *P*-values are based on the Kruskal-Wallis test followed by Dunn’s post-hoc test for multiple comparison.

**Additional file 5: Figure S5**. P21 and P16 senescence markers are increased in isolated pulmonary arteries of patients with idiopathic pulmonary fibrosis (IPF) and pulmonary hypertension (PH) associated to IPF. mRNA expression of P21 and P16 (A, B) in isolated pulmonary arteries (70-500µm of internal diameter) from control (n=20), IPF (n=20) and IPF + PH (n=20) patients. (C, D) Correlation between P21 and P16 mRNA expression with IL-11 protein expression in pulmonary arteries from IPF + PH patients. (E) Co-immunofluorescence of P21 and IL-11 in pulmonary arteries from IPF + PH patients. Scale bar: 30 µm. Data are presented as scatter dot blot with median and interquartile range values. *P*-values are based on the Kruskal-Wallis test and Dunn’s post-hoc test for multiple comparison. (M) Spearman ρ correlation of IL-11 and P21/P16 expression in isolated pulmonary arteries from PH+IPF.

**Additional file 6: Figure S6**. P21 co-localize in IL-11 positive cells in pulmonary arteries from mice treated with rmIL-11 and bleomycin. (A) A total of 100µg/Kg/day of rmIL-11 or saline (control) was subcutaneously administered during 21 days in tie2-GFP mice. At day 21 lungs were fixed. (B) Wild-type (WT) siRNA(-) tie2-GFP mice and IL-11-KO siRNA-IL-11 tie2-GFP mice received a single intratracheal dose of bleomycin (1.5 U/kg) on day 1 during 14 days. At day 14 lung were fixed. (A, B) Lungs were immunostained with P21 and IL-11 antibodies followed by secondary antibodies with FITC/Rhodamine fluorescence probes. Representative images are showed.

**Additional file 7.** Figure legends.

**Additional file 8: Table S1.** Clinical data of control subjects. **Table S2.** Clinical data of IPF patients. **Table S3.** Clinical data of IPF+PH patients. **Table S4.** Clinical differences between IPF and IPF+PH patients.

**Additional file 9.** Supplementary figures.

## Data Availability

The datasets used and/or analyzed during the current study are available from the corresponding author upon reasonable request.

## Abbreviations

CLD: Chronic lung disease
EnMT: Endothelial to mesenchymal transition
HPAEC: Human pulmonary artery endothelial cells
HPASMC: Human pulmonary artery smooth muscle cells
IPF: Idiopathic pulmonary fibrosis
PH: Pulmonary hypertension
RVSP: Right ventricular systolic pressure
SASP: Senescence-associated secretory phenotype
