# Supplementary figures and images for: IL-11 system participates in pulmonary artery remodeling and hypertension in pulmonary fibrosis
Source: Respir Res. 2022 Nov 15;23:313. doi: 10.1186/s12931-022-02241-0 (PMC9664718; doi:10.1186/s12931-022-02241-0)

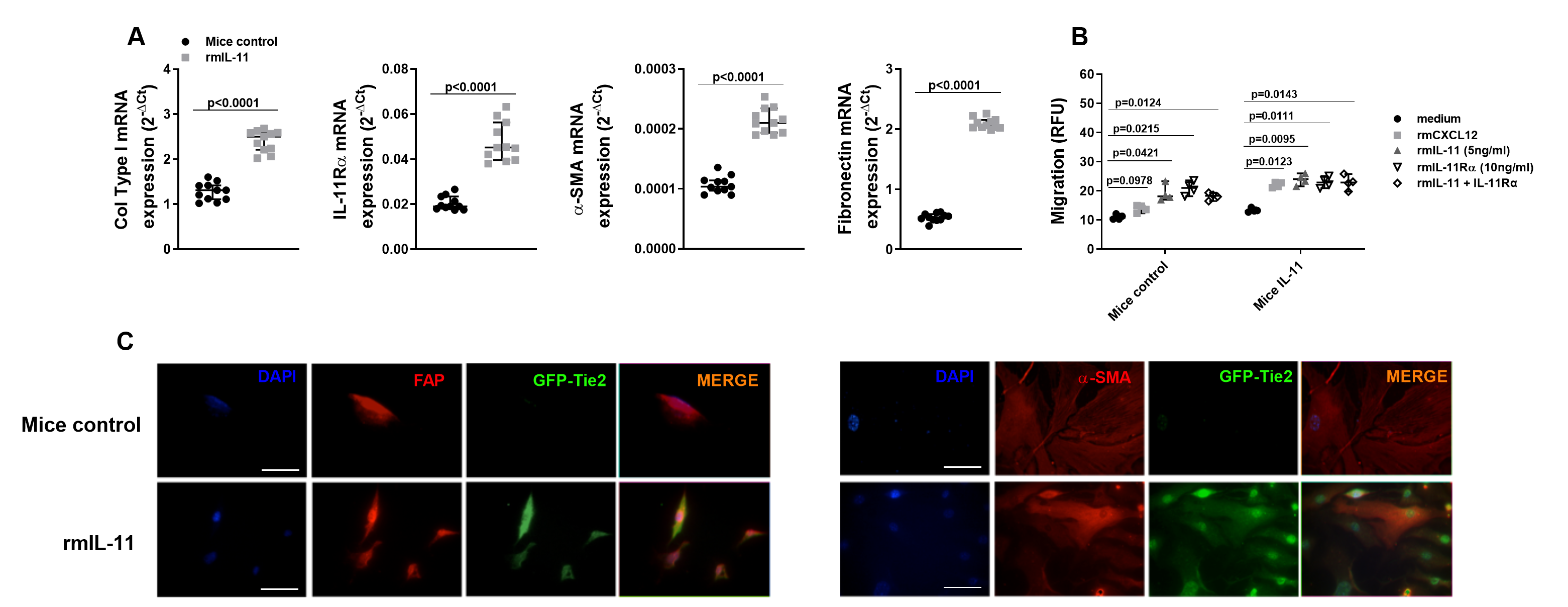

Supplement: Supplementary file 1 — Additional file 1: Figure S1. mrIL-11 induces endothelial to mesenchymal transition (EnMT) in vivo, promoting parenchymal myofibroblast-like cells of endothelial origin. A total of 100µg/Kg/day of rmIL-11 (n=11) or saline (n=11) was subcutaneously administered during 21 days. At day 21 parenchymal lug fibroblast were isolated and cultured at passage 1. (A) The expression of extracellular matrix proteins, αSMA and IL-11Rα was measured by quantitative PCR (qPCR) as 2−ΔCt. (B) Isolated lung fibroblasts from sham control or mrIL-11 treated animals studied to analyse the migratory capacity following basal, rmCXCL12, rmIL-11, rmIL-11Rα or its combination. (C) Co-immunofluorescence analysis of FAP/tie2-GFP and αSMA/ tie2-GFP of isolated parenchymal fibroblasts. Scale bar: 10µm. Data are presented as scatter dot blot with median and interquartile range values. P-values are based on the (A) Mann Whitney or (B) Kruskal-Wallis test followed by Dunn’s post-hoc test for multiple comparison. [file 12931_2022_2241_MOESM1_ESM.tif]

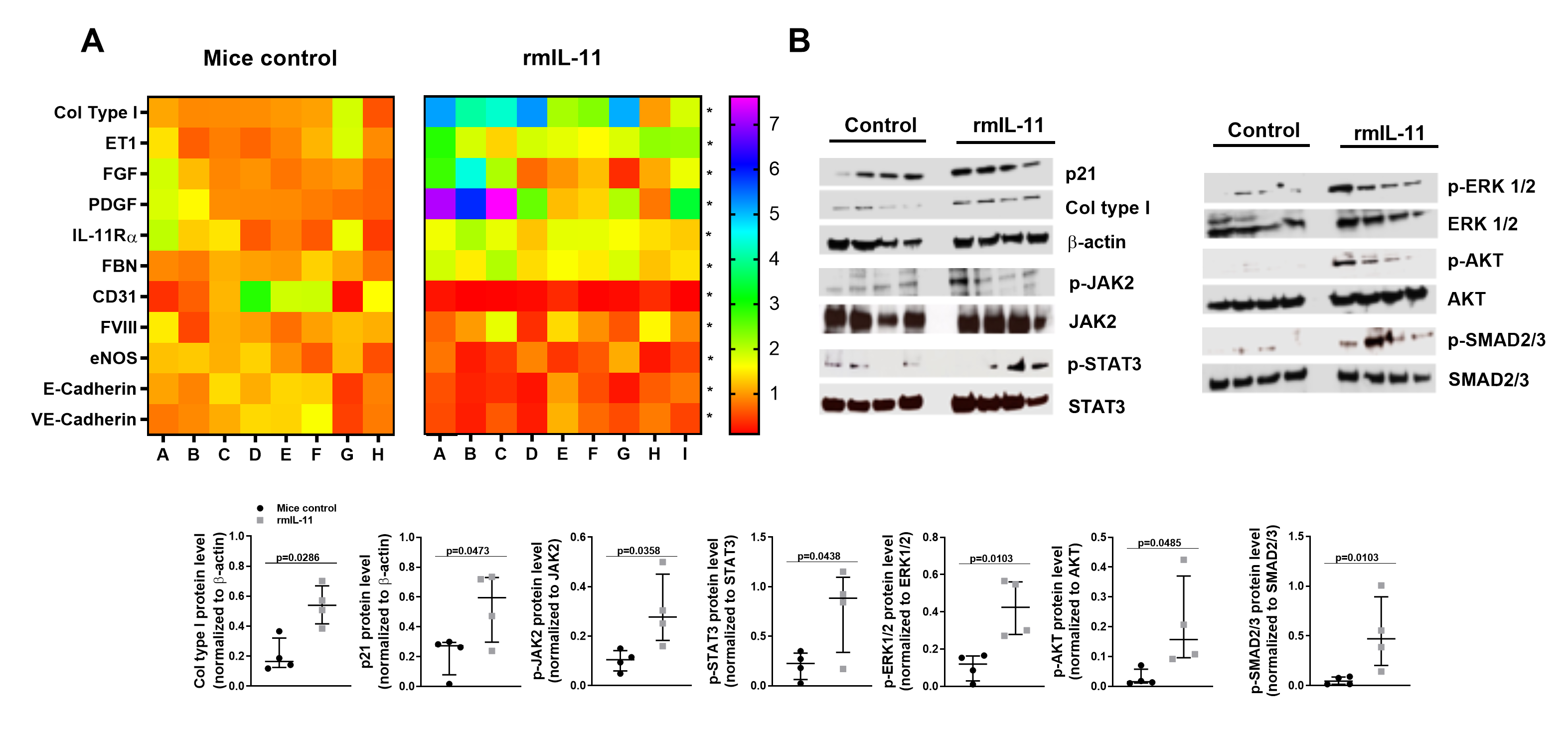

Supplement: Supplementary file 2 — Additional file 2: Figure S2. mrIL-11 induces lung tissue remodeling increasing profibrotic markers and reducing endothelial cell markers. A total of 100µg/Kg/day of rmIL-11 (n=11) or saline (n=11) was subcutaneously administered during 21 days. At day 21 lung homogenates were processed to (A) measure profibrotic and endothelial cell markers presented by heat map representation of mRNA transcripts of different genes and measured by quantitative PCR (qPCR) as 2−ΔCt. *P < 0.05 vs sham controls (B) Intracellular signalling markers, collagen type I and senescence P21 marker protein expression by western blotting. Data are shown as the ratio compared to β-actin or non-phosphorylated protein as indicate. Data are presented as scatter dot blot with median and interquartile range values. P-values are based on the Mann Whitney test. [file 12931_2022_2241_MOESM2_ESM.tif]

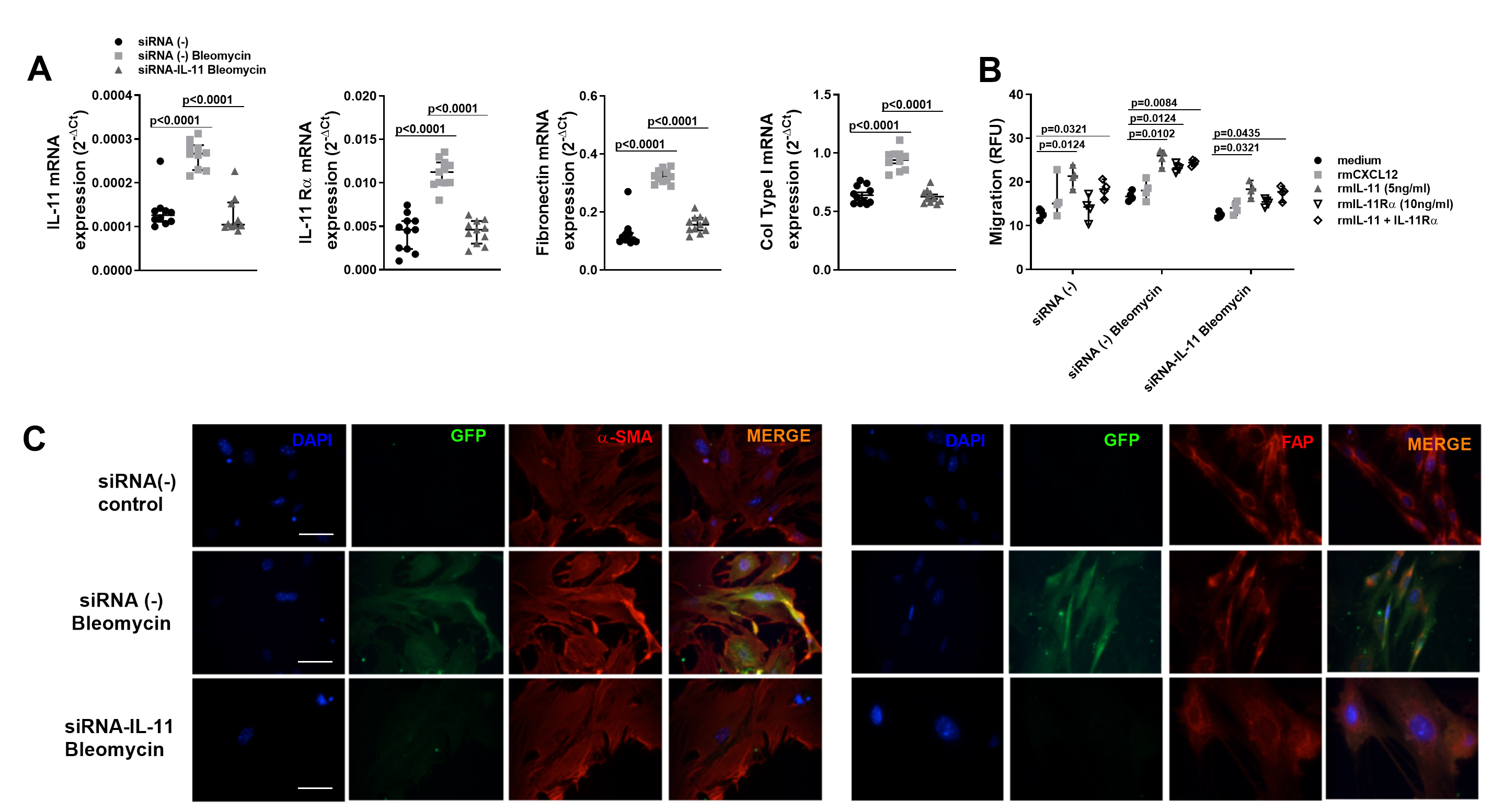

Supplement: Supplementary file 3 — Additional file 3: Figure S3. SiRNA-IL-11 transiently transfection reduces bleomycin-induced endothelial to mesenchymal transition (EnMT) in vivo, suppressing parenchymal myofibroblast-like cells of endothelial origin. Wild-type (WT) siRNA(-) tie2-GFP mice and IL-11-KO siRNA-IL-11 tie2-GFP mice received a single intratracheal dose of bleomycin (1.5 U/kg) on day 1 (n = 11) during 14 days. At day 14 parenchymal lug fibroblast were isolated and cultured at passage 1. (A) The expression of extracellular matrix proteins, IL-11 and IL-11Rα was measured by quantitative PCR (qPCR) as 2−ΔCt. (B) Isolated lung fibroblasts from sham siRNA(-) control, siRNA(-) bleomycin and siRNA-IL-11 bleomycin treated animals were studied to analyse the migratory capacity following the stimulation with basal medium, rmCXCL12, rmIL-11, rmIL-11Rα or its combination. (C) Co-immunofluorescence analysis of FAP/tie2-GFP and αSMA/ tie2-GFP of isolated parenchymal fibroblasts. Scale bar: 10µm. Data are presented as scatter dot blot with median and interquartile range values. P-values are based on the Kruskal-Wallis test followed by Dunn’s post-hoc test for multiple comparison. [file 12931_2022_2241_MOESM3_ESM.tif]

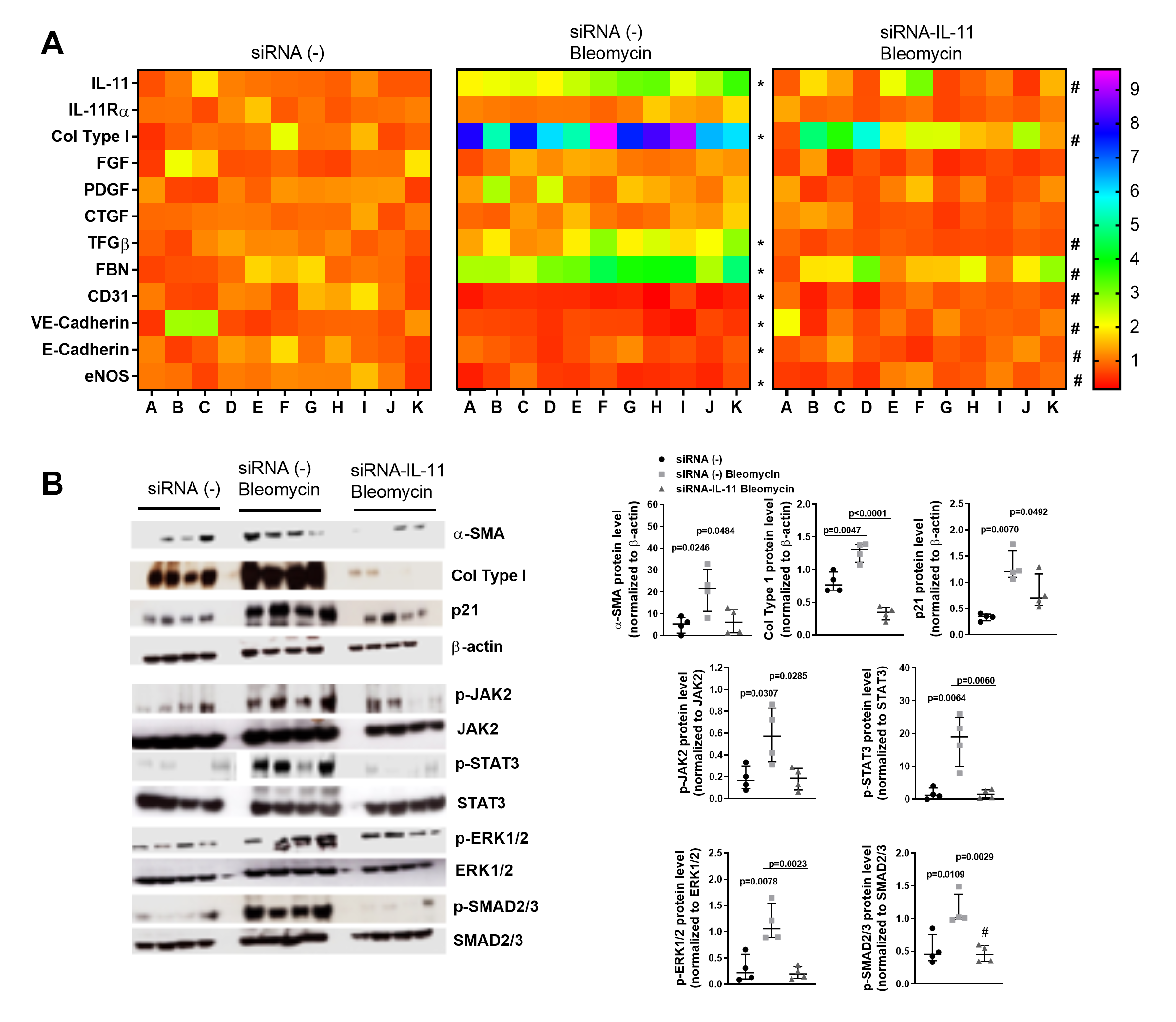

Supplement: Supplementary file 4 — Additional file 4: Figure S4. SiRNA-IL-11 transiently transfection attenuates bleomycin-induced lung tissue remodeling reducing profibrotic markers and increasing endothelial cell markers. Wild-type (WT) siRNA(-) tie2-GFP mice and IL-11-KO siRNA-IL-11 tie2-GFP mice received a single intratracheal dose of bleomycin (1.5 U/kg) on day 1 (n = 11) during 14 days. At day 14 lung homogenates were processed to (A) measure profibrotic and endothelial cell markers presented by heat map representation of mRNA transcripts of different genes and measured by quantitative PCR (qPCR) as 2−ΔCt. *P < 0.05 vs sham siRNA(-) controls; #P <0.05 vs siRNA(-) bleomycin mice group. (B) Intracellular signalling markers, collagen type I and senescence P21 marker protein expression by western blotting. Data are shown as the ratio compared to β-actin or non-phosphorylated protein as indicate. Representative blots are sowed. Data are presented as scatter dot blot with median and interquartile range values. P-values are based on the Kruskal-Wallis test followed by Dunn’s post-hoc test for multiple comparison. [file 12931_2022_2241_MOESM4_ESM.tif]

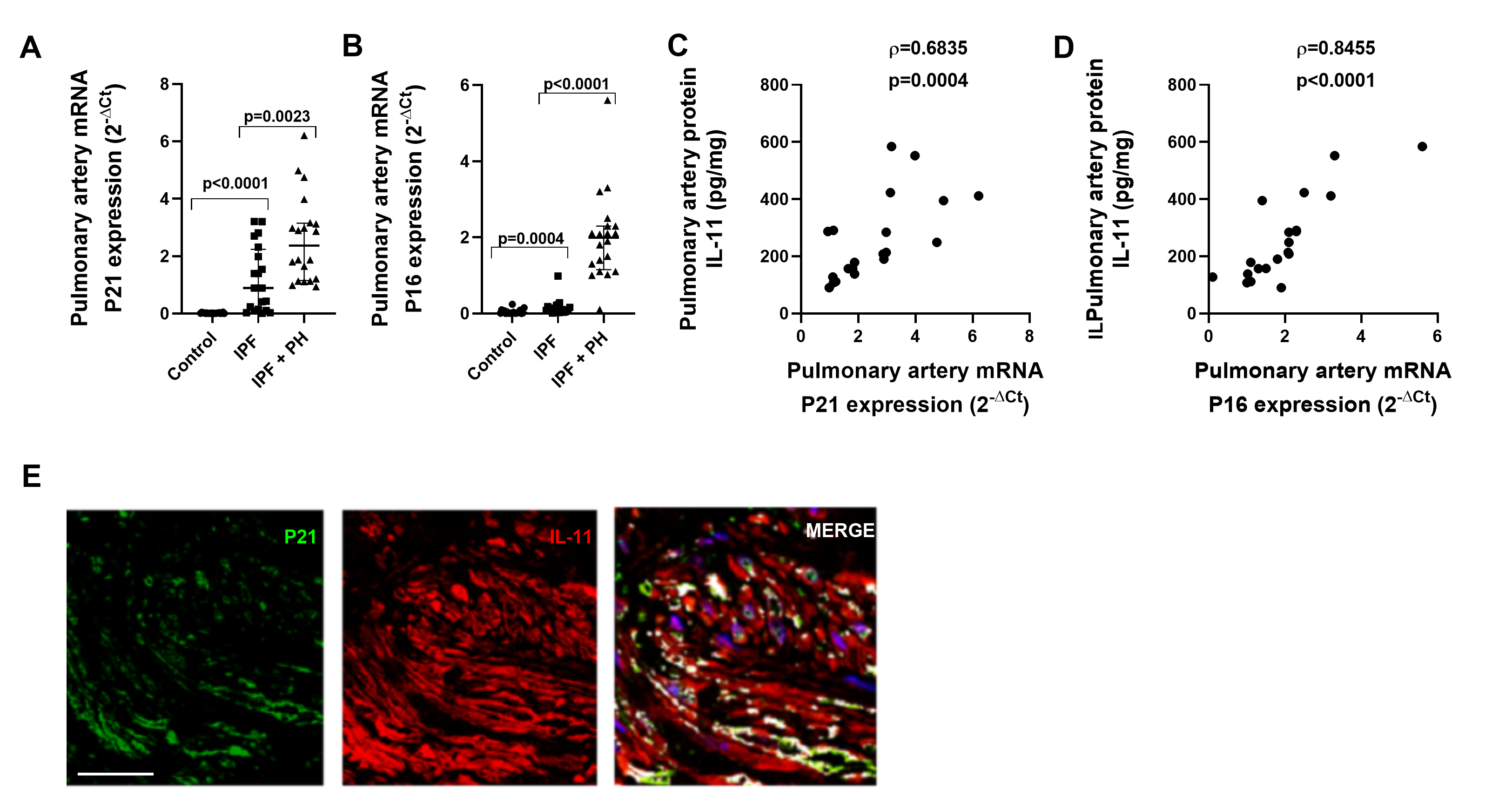

Supplement: Supplementary file 5 — Additional file 5: Figure S5. P21 and P16 senescence markers are increased in isolated pulmonary arteries of patients with idiopathic pulmonary fibrosis (IPF) and pulmonary hypertension (PH) associated to IPF. mRNA expression of P21 and P16 (A, B) in isolated pulmonary arteries (70-500µm of internal diameter) from control (n=20), IPF (n=20) and IPF + PH (n=20) patients. (C, D) Correlation between P21 and P16 mRNA expression with IL-11 protein expression in pulmonary arteries from IPF + PH patients. (E) Co-immunofluorescence of P21 and IL-11 in pulmonary arteries from IPF + PH patients. Scale bar: 30 µm. Data are presented as scatter dot blot with median and interquartile range values. P-values are based on the Kruskal-Wallis test and Dunn’s post-hoc test for multiple comparison. (M) Spearman ρ correlation of IL-11 and P21/P16 expression in isolated pulmonary arteries from PH+IPF. [file 12931_2022_2241_MOESM5_ESM.tif]

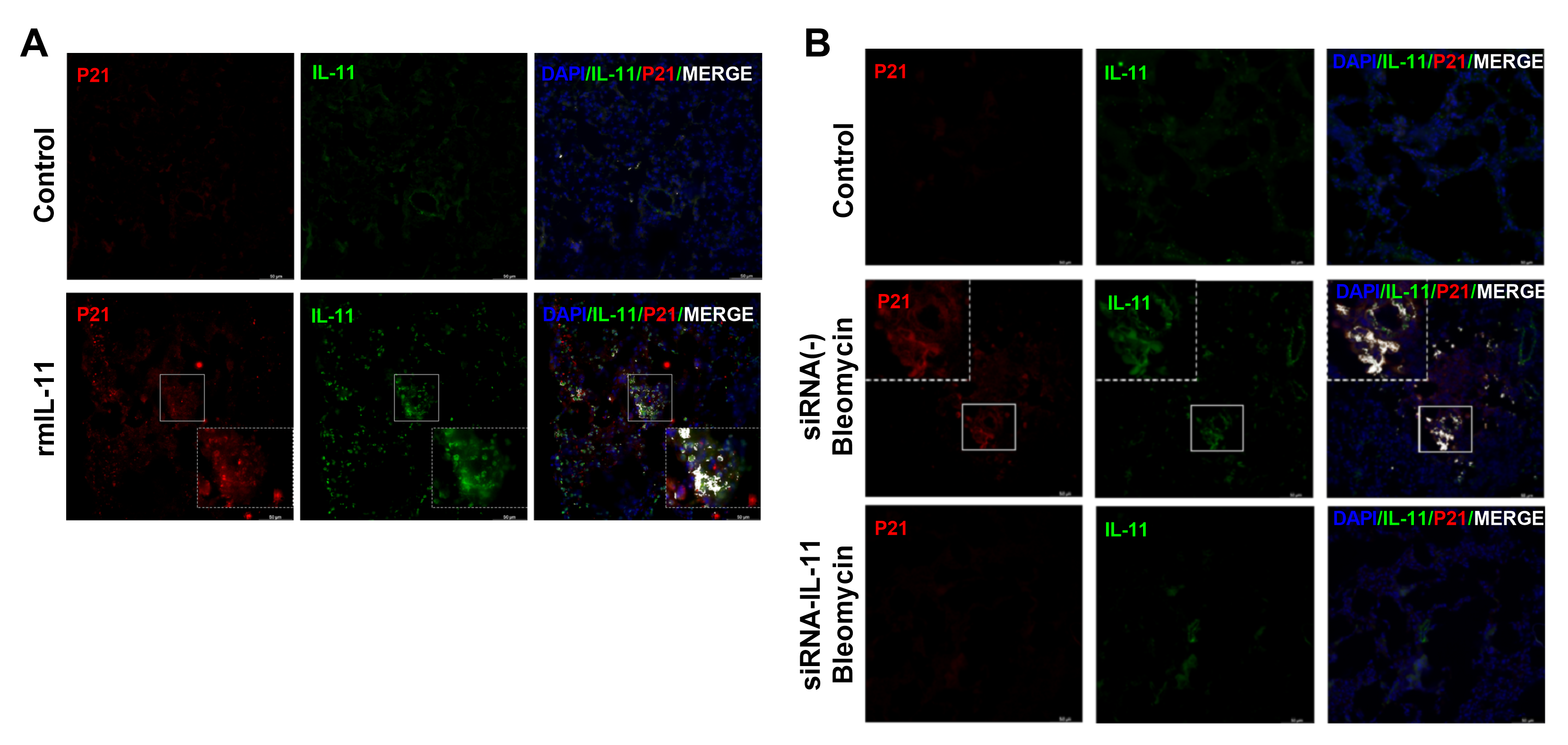

Supplement: Supplementary file 6 — Additional file 6: Figure S6. P21 co-localize in IL-11 positive cells in pulmonary arteries from mice treated with rmIL-11 and bleomycin. (A) A total of 100µg/Kg/day of rmIL-11 or saline (control) was subcutaneously administered during 21 days in tie2-GFP mice. At day 21 lungs were fixed. (B) Wild-type (WT) siRNA(-) tie2-GFP mice and IL-11-KO siRNA-IL-11 tie2-GFP mice received a single intratracheal dose of bleomycin (1.5 U/kg) on day 1 during 14 days. At day 14 lung were fixed. (A, B) Lungs were immunostained with P21 and IL-11 antibodies followed by secondary antibodies with FITC/Rhodamine fluorescence probes. Representative images are showed. [file 12931_2022_2241_MOESM6_ESM.tif]
