# Supplementary material for: IL-11 system participates in pulmonary artery remodeling and hypertension in pulmonary fibrosis
Source: Respir Res. 2022 Nov 15;23:313. doi: 10.1186/s12931-022-02241-0 (PMC9664718; doi:10.1186/s12931-022-02241-0)
Supplement: Supplementary file 8 — Additional file 8: Table S1. Clinical data of control subjects. Table S2. Clinical data of IPF patients. Table S3. Clinical data of IPF+PH patients. Table S4. Clinical differences between IPF and IPF+PH patients. [file 12931_2022_2241_MOESM8_ESM.docx]

**SUPPLEMENTARY TABLES**

**Table S1. Clinical data of control subjects**. CAV: cerebrovascular disease. F: female; M: male; N/A: not available.

| **ID** | **Age (years)** | **Sex (M/F)** | **Smoker (yes/no)** | **Cause of death** |
| --- | --- | --- | --- | --- |
| **H338** | 42 | F | Yes | Intracranial hemorrhage |
| **H345** | 55 | M | No | CVA |
| **H358** | 43 | F | Yes | Cerebrovascular/stroke |
| **H359** | 62 | F | N/A | Trafic accident |
| **H361** | 52 | F | N/A | Trafic accident |
| **H363** | 42 | M | N/A | Trafic accident |
| **H369** | 34 | M | Yes | Cerebrovascular stroke/intracranial hemorrhage |
| **H373** | 55 | F | Yes | Acute myocardial infarction |
| **H374** | 61 | F | Yes | Intracranial hemorrhage |
| **H376** | 66 | M | Yes | Subarachnoid hemorrhage |
| **H377** | 53 | M | No | Trafic accident |
| **H395** | 57 | M | N/A | Trafic accident |
| **H404** | 34 | M | N/A | Trafic accident |
| **H414** | 67 | F | No | Anoxia of brain |
| **H418** | 38 | F | No | Intracranial hemorrhage |
| **H421** | 34 | F | N/A | Trafic accident |
| **H429** | 62 | M | Yes | CVA |
| **H437** | 35 | M | Yes | CVA |
| **H439** | 45 | M | Yes | Intracranial hemorrhage |
| **H447** | 62 | M | N/A | Trafic accident |
| **H453** | 54 | F | N/A | Trafic accident |

**Table S2. Clinical data of IPF patients.** % pred, % predicted; DLco, diffusion capacity of the lung for carbon monoxide; F: female; FEV1, forced expiratory volume in 1 s; FVC, forced vital capacity; N/A: not available; M: male; Pack-year, 1 year of smoking 20 cigarettes per day; PaO2, arterial blood oxygen tension; 6MWT: 6 minutes walking test.

| **ID** | **Age** | **Sex (M/F)** | **Smoker (pack/year)** | **FVC %, pred** | **FEV1 %, pred** | **DLCO%, pred** | **PaO2, mmHg** | **6MWT** | **mPAP (mmHg/L/min)** | **Antifibrotic drug** |
| --- | --- | --- | --- | --- | --- | --- | --- | --- | --- | --- |
| **IPF1** | 63 | M | 40 | 57 | 71 | 47 | 65 | 178 | 12 | Pirfenidone |
| **IPF2** | 78 | M | 0 | 62 | 75 | 52 | 49 | 245 | 10 | Pirfenidone |
| **IPF3** | 52 | F | 40 | 67 | 82 | 39 | 52 | 356 | 18 | Nintedanib/Pirfenidone |
| **IPF4** | 41 | M | 0 | 98 | 70 | 46 | 67 | 498 | 11 | Ninedanib |
| **IPF5** | 56 | M | 35 | 85 | 90 | 68 | 55 | 185 | 15 | Pirfenidone |
| **IPF6** | 50 | M | 21 | 57 | 84 | 26 | 56 | 183 | 9 | Ninedanib |
| **IPF7** | 72 | M | 22 | 89 | 82 | 79 | 76 | 451 | 17 | Pirfenidone |
| **IPF8** | 55 | F | 0 | 74 | 62 | 56 | 74 | 356 | 15 | Pirfenidone |
| **IPF9** | 64 | M | 35 | 72 | 74 | 59 | 66 | 298 | 14 | Nintedanib/Pirfenidone |
| **IPF10** | 62 | F | 21 | 71 | 72 | 48 | 62 | 224 | 13 | Nintedanib/Pirfenidone |
| **IPF11** | 65 | M | 32 | 68 | 62 | 51 | 72 | 341 | 16 | Ninedanib |
| **IPF12** | 64 | M | 0 | 67 | 73 | 39 | 75 | 321 | 17 | Pirfenidone |
| **IPF13** | 61 | F | 22 | 58 | 59 | 42 | 68 | 256 | 13 | Pirfenidone |
| **IPF14** | 68 | M | 26 | 47 | 67 | 35 | 49 | 265 | 12 | Pirfenidone |
| **IPF15** | 60 | M | 31 | 74 | 75 | 38 | 56 | 354 | 18 | Ninedanib |
| **IPF16** | 58 | M | 25 | 69 | 62 | 36 | 74 | 284 | 11 | Ninedanib |
| **IPF17** | 55 | M | 28 | 68 | 81 | 59 | 55 | 265 | 12 | Ninedanib |
| **IPF18** | 69 | M | 30 | 43 | 92 | 47 | 62 | 211 | 11 | Pirfenidone |
| **IPF19** | 59 | M | 0 | 58 | 76 | 42 | 65 | 186 | 19 | Pirfenidone |
| **IPF20** | 61 | M | 33 | 65 | 64 | 41 | 64 | 265 | 15 | Ninedanib |
| **IPF21** | 64 | M | 32 | 78 | 68 | 42 | 50 | 287 | 17 | Ninedanib |

**Table S3. Clinical data of IPF+PH patients.** % pred, % predicted; DLco, diffusion capacity of the lung for carbon monoxide; F: female; FEV1, forced expiratory volume in 1 s; FVC, forced vital capacity; N/A: not available; M: male; mPAP: mean pulmonary artery pressure; Pack-year, 1 year of smoking 20 cigarettes per day; PaO2, arterial blood oxygen tension; 6MWT: 6 minutes walking test.

| **ID** | **Age (years)** | **Sex (M/F)** | **Smokers (pack/year)** | **FVC %, pred** | **FEV1 %, pred** | **DLCO%, pred** | **PaO2, mmHg** | **6MWT** | **mPAP (mmHg/L/min)** | **Cardiac output (L/min)** | **Cardiac index (L/min/m^2^)** | **PVR (mmHg/L/min)** | **PAWP, mmHg** | **RAP, mmHg** | **Antifibrotic drug** |
| --- | --- | --- | --- | --- | --- | --- | --- | --- | --- | --- | --- | --- | --- | --- | --- |
| **HP1** | 65 | F | 35 | 49,7 | 54 | 22 | 66 | 245 | 29 | 5.8 | 2.9 | 3.2 | 10.2 | 7.8 | Pirfenidone |
| **HP2** | 62 | M | 10 | 53 | 62 | 32 | 51 | 185 | 47 | 4.8 | 2.5 | 3.9 | 9.6 | 6.1 | Pirfenidone |
| **HP3** | 74 | M | 21 | 53,4 | 95 | 36 | 36 | 456 | 35 | 5.3 | 2.6 | 3.5 | 8.5 | 6.4 | Nintedanib/pirfenidone |
| **HP4** | 71 | M | 0 | 109 | 92 | 66 | 45 | 354 | 28 | 5.1 | 3.5 | 3.1 | 6.5 | 7.2 | Nintedanib |
| **HP5** | 64 | M | 24 | 84,4 | 75 | 74 | 56 | 146 | 33 | 5.6 | 3.21 | 3.7 | 9.2 | 7.4 | Pirfenidone |
| **HP6** | 55 | F | 30 | 49,9 | 74 | 17 | 42 | 186 | 42 | 4.9 | 2.6 | 3.8 | 10.5 | 8.4 | Pirfenidone |
| **HP7** | 68 | M | 25 | 115 | 95 | 96 | 79 | 342 | 46 | 4.8 | 2.7 | 3.4 | 11.3 | 10.2 | Nintedanib/pirfenidone |
| **HP8** | 49 | M | 4 | 69 | 62 | 46 | 58 | 465 | 51 | 4.1 | 2.3 | 3.8 | 9.5 | 6.1 | Nintedanib/pirfenidone |
| **HP9** | 67 | F | 30 | 56 | 64 | 55 | 64 | 226 | 32 | 5.1 | 3.6 | 3.2 | 10.6 | 7.8 | Nintedanib |
| **HP10** | 59 | F | 26 | 51 | 95 | 42 | 59 | 296 | 41 | 4.7 | 3.5 | 3.4 | 9.5 | 7.5 | Pirfenidone |
| **HP11** | 68 | F | 28 | 59,1 | 65 | 39,6 | 48 | 312 | 29 | 5.4 | 3.6 | 3.1 | 8.5 | 9.5 | Nintedanib |
| **HP12** | 72 | M | 10 | 55 | 62 | 38 | 46 | 345 | 30 | 5.9 | 3.4 | 2.9 | 10.5 | 10.4 | Nintedanib |
| **HP13** | 62 | M | 28 | 40 | 52 | 41 | 44 | 265 | 44 | 4.7 | 2.8 | 3.9 | 7.7 | 6.9 | Pirfenidone |
| **HP14** | 57 | M | 25 | 52 | 85 | 26 | 46 | 238 | 26 | 5.7 | 3.6 | 2.8 | 10.5 | 10.5 | Pirfenidone |
| **HP15** | 59 | M | 29 | 67 | 74 | 29 | 65 | 281 | 29 | 5.4 | 3.7 | 3.0 | 11 | 9.2 | Pirfenidone |
| **HP16** | 64 | M | 24 | 51 | 62 | 20 | 55 | 365 | 38 | 5.2 | 2.8 | 3.5 | 9.2 | 8.2 | Pirfenidone |
| **HP17** | 65 | M | 30 | 56 | 66 | 24 | 56 | 215 | 46 | 4.9 | 2.5 | 4.2 | 11.3 | 11.2 | Pirfenidone |
| **HP18** | 59 | M | 25 | 43 | 67 | 33 | 62 | 265 | 55 | 4.2 | 2.3 | 4.3 | 7.5 | 8.2 | Nintedanib |
| **HP19** | 62 | F | 5 | 33 | 98 | 28 | 38 | 259 | 42 | 5.4 | 2.7 | 3.8 | 8.6 | 7.6 | Nintedanib |
| **HP20** | 64 | F | 0 | 55 | 62 | 21 | 46 | 168 | 43 | 4.9 | 2.9 | 3.6 | 9.3 | 8.1 | Pirfenidone |
| **HP21** | 61 | F | 34 | 61 | 61 | 34 | 51 | 298 | 46 | 5.3 | 3.1 | 3.3 | 10.2 | 9.3 | Pirfenidone |

**Table S4. Clinical differences between IPF and IPF+PH patients.** % pred, % predicted; DLco, diffusion capacity of the lung for carbon monoxide; F: female; FEV1, forced expiratory volume in 1 s; FVC, forced vital capacity; N/A: not available; M: male; mPAP: mean pulmonary artery pressure; Pack-year, 1 year of smoking 20 cigarettes per day; PaO2, arterial blood oxygen tension; 6MWT: 6 minutes walking test.

| **Clinical data** | **IPF** | **IPF + PH** | **Statistical exact p values** |
| --- | --- | --- | --- |
| Age (years, median IQR) | 61 [55.5-64.5] | 64 [59-67.5] | 0.2674 |
| Sex (*n*, M/F), | 17M/3F | 13M/7F | 0.0891 |
| Smokers (pack/year, median IQR) | 26 [10.5-32.5] | 25 [10-29.5 ] | 0.5358 |
| FVC %, pred, median IQR | 68 [58-74] | 55 [50.45-64] | 0.0077* |
| FEV1 %, pred, median IQR | 73 [65.5-81.5] | 66 [62-88.5] | 0.4552 |
| DLCO%, pred, median IQR | 46 [39-54] | 34 [25-44] | 0.0098* |
| PaO2, mmHg, median IQR | 64 [55-70] | 51 [45.5-61.5] | 0.0036* |
| 6MWT, median IQR | 265 [217.5-347.5] | 265 [220.5-343.5] | 0.9058 |
| mPAP (mmHg/L/min), median IQR | 14 [11.5-17] | 41 [29.5-46] | <0.0001* |
| Antifibrotic drug (pirfenidone (P), nintedanib (N), *n* | 13P/8N | 15P/9N | 0.9821 |
