# Supplementary material for: IL-11 system participates in pulmonary artery remodeling and hypertension in pulmonary fibrosis
Source: Respir Res. 2022 Nov 15;23:313. doi: 10.1186/s12931-022-02241-0 (PMC9664718; doi:10.1186/s12931-022-02241-0)

Figure 5

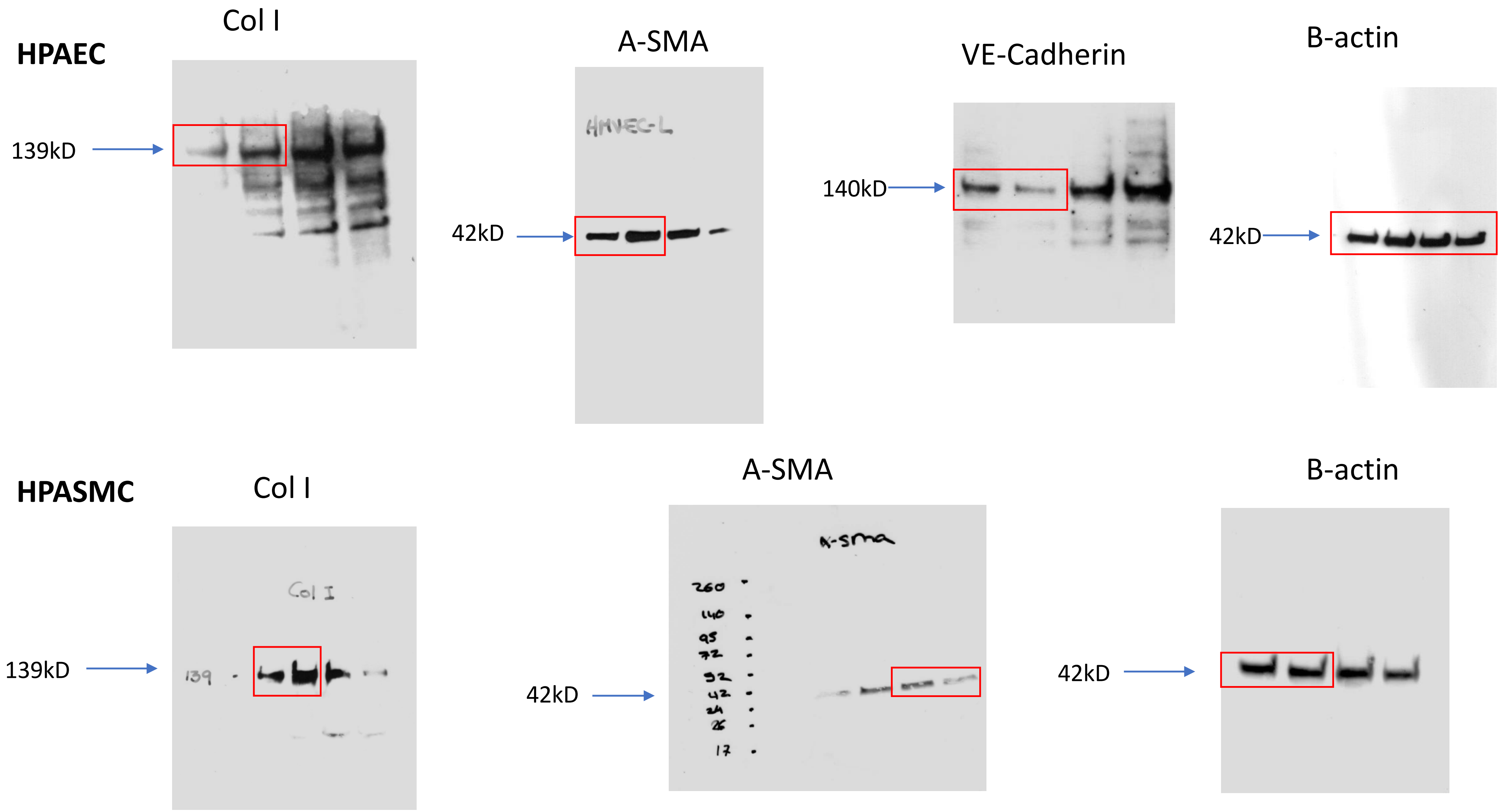
HPAEC
Col I
139kD
A-SMA
42kD
VE-Cadherin
140kD
B-actin
42kD
HPASMC
Col I
139kD
A-SMA
42kD
B-actin
42kD

Figure 6

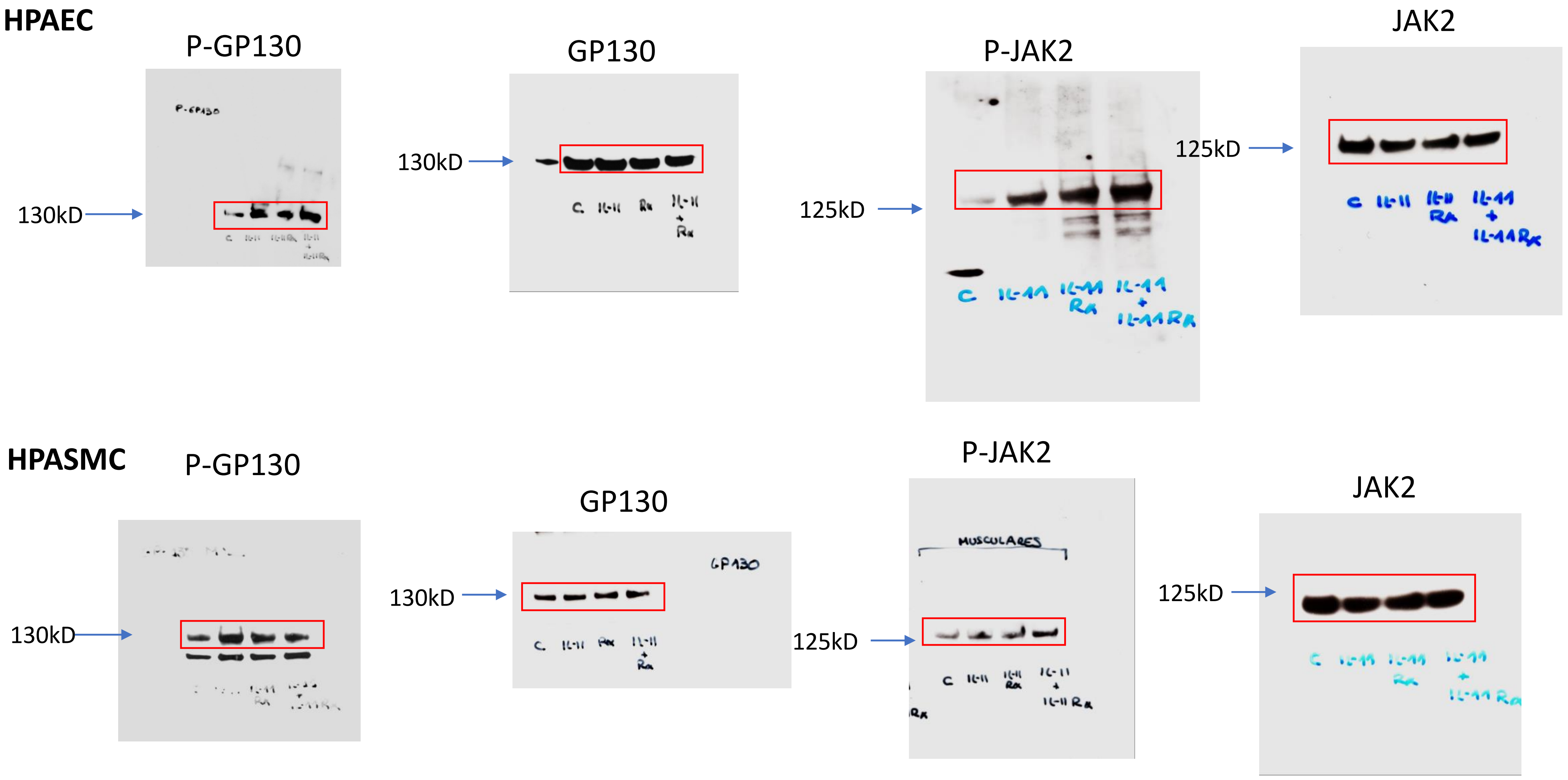
HPAEC
P-GP130
130kD
GP130
130kD
P-JAK2
125kD
JAK2
125kD
HPASMC
P-GP130
130kD
GP130
130kD
P-JAK2
125kD
JAK2
125kD

Figure 6

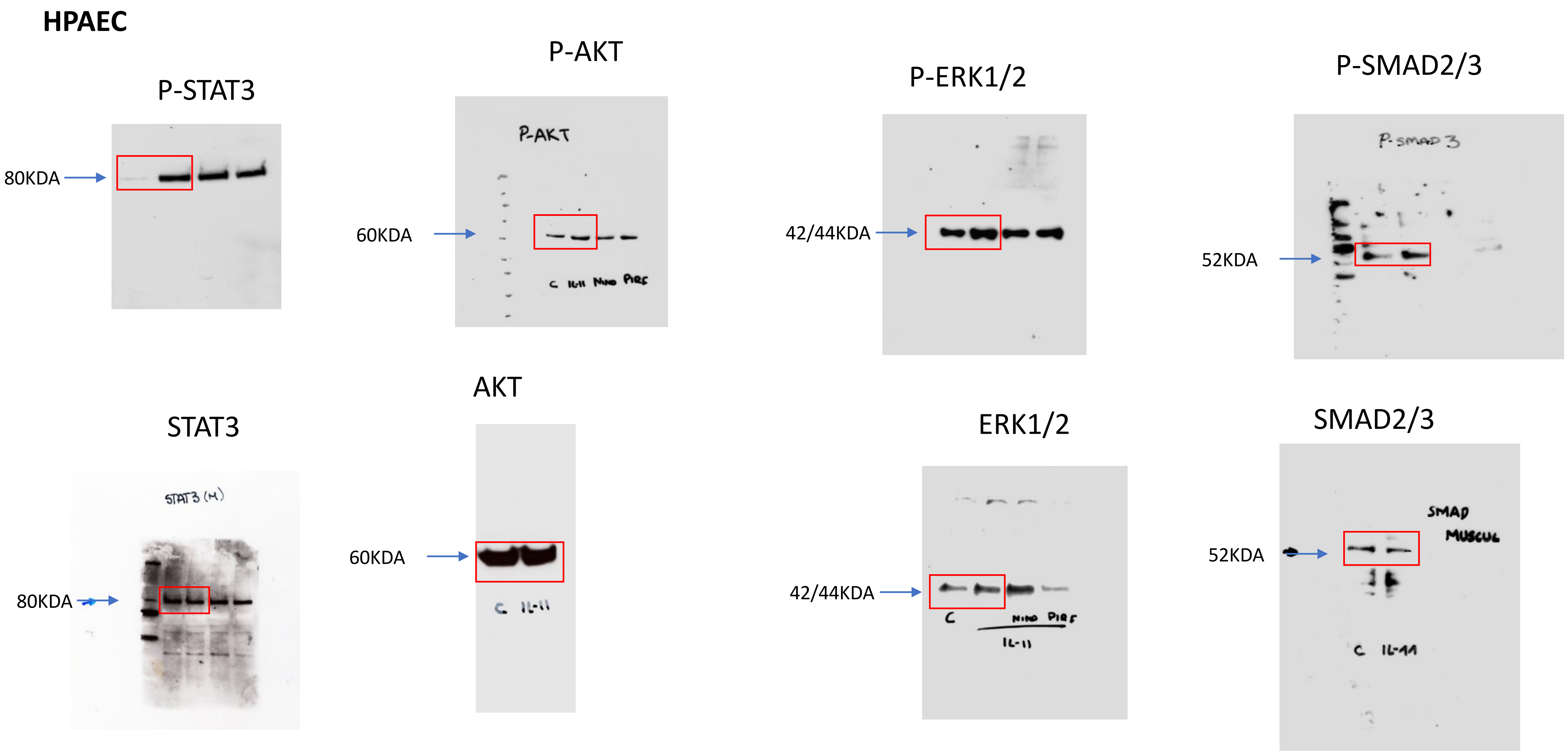
HPAEC
P-STAT3
80KDA
P-AKT
60KDA
P-ERK1/2
42/44KDA
P-SMAD2/3
52KDA
STAT3
80KDA
AKT
60KDA
ERK1/2
42/44KDA
SMAD2/3
52KDA

Figure 6

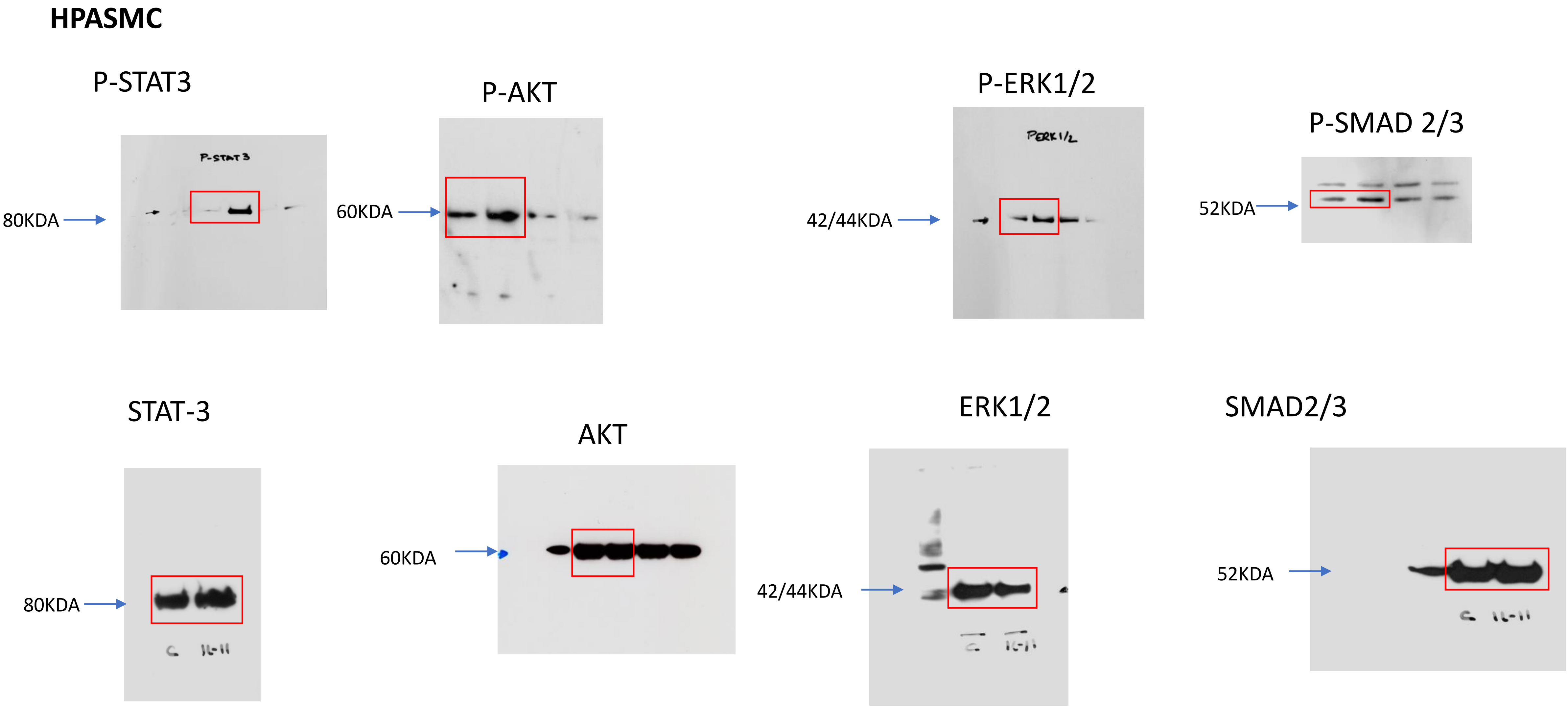
HPASMC
P-STAT3
P-AKT
P-ERK1/2
P-SMAD 2/3
80KDA
60KDA
42/44KDA
52KDA
STAT-3
AKT
ERK1/2
SMAD2/3
80KDA
60KDA
42/44KDA
52KDA

Figure 8

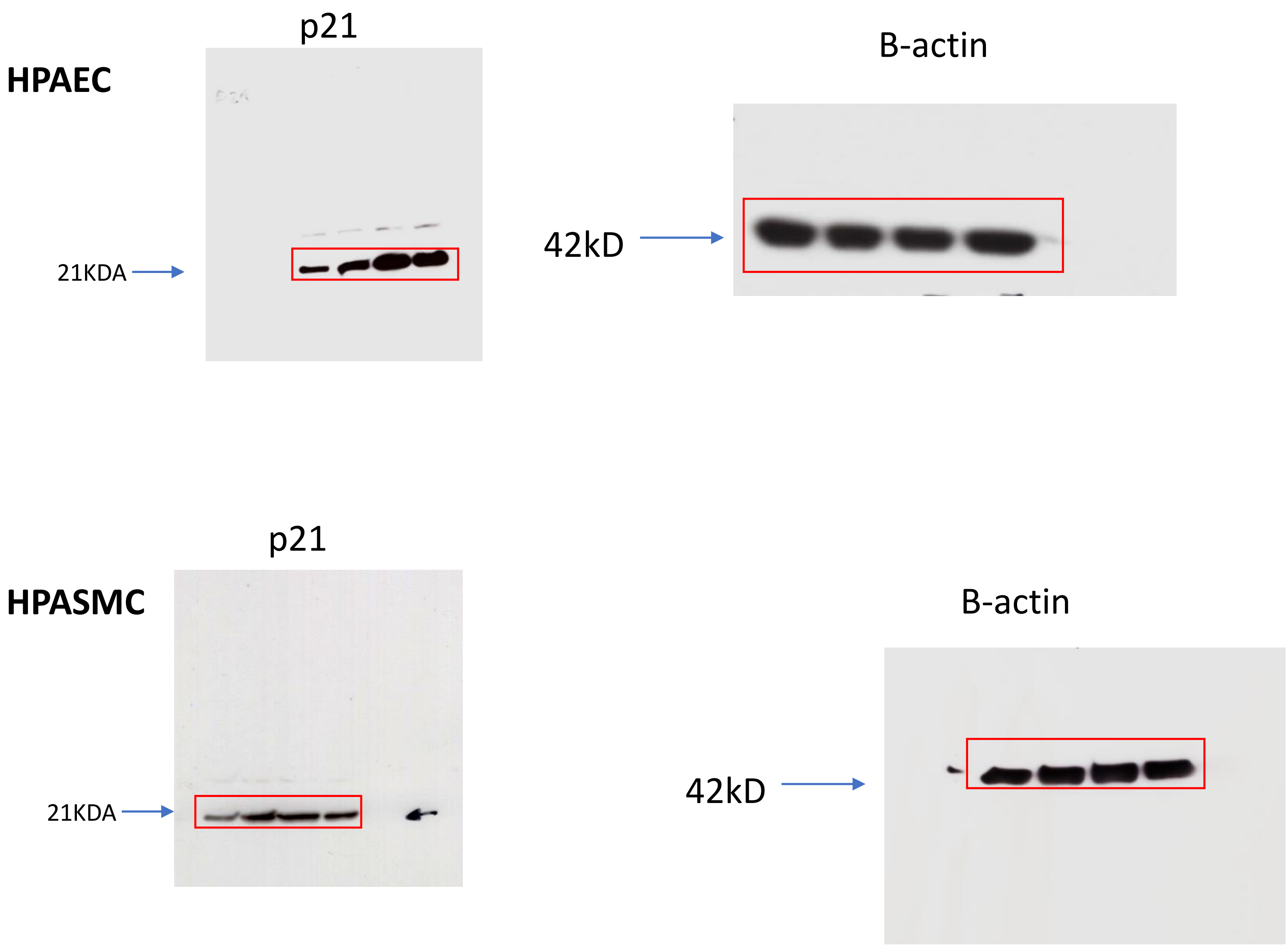

Supplementary figure S2

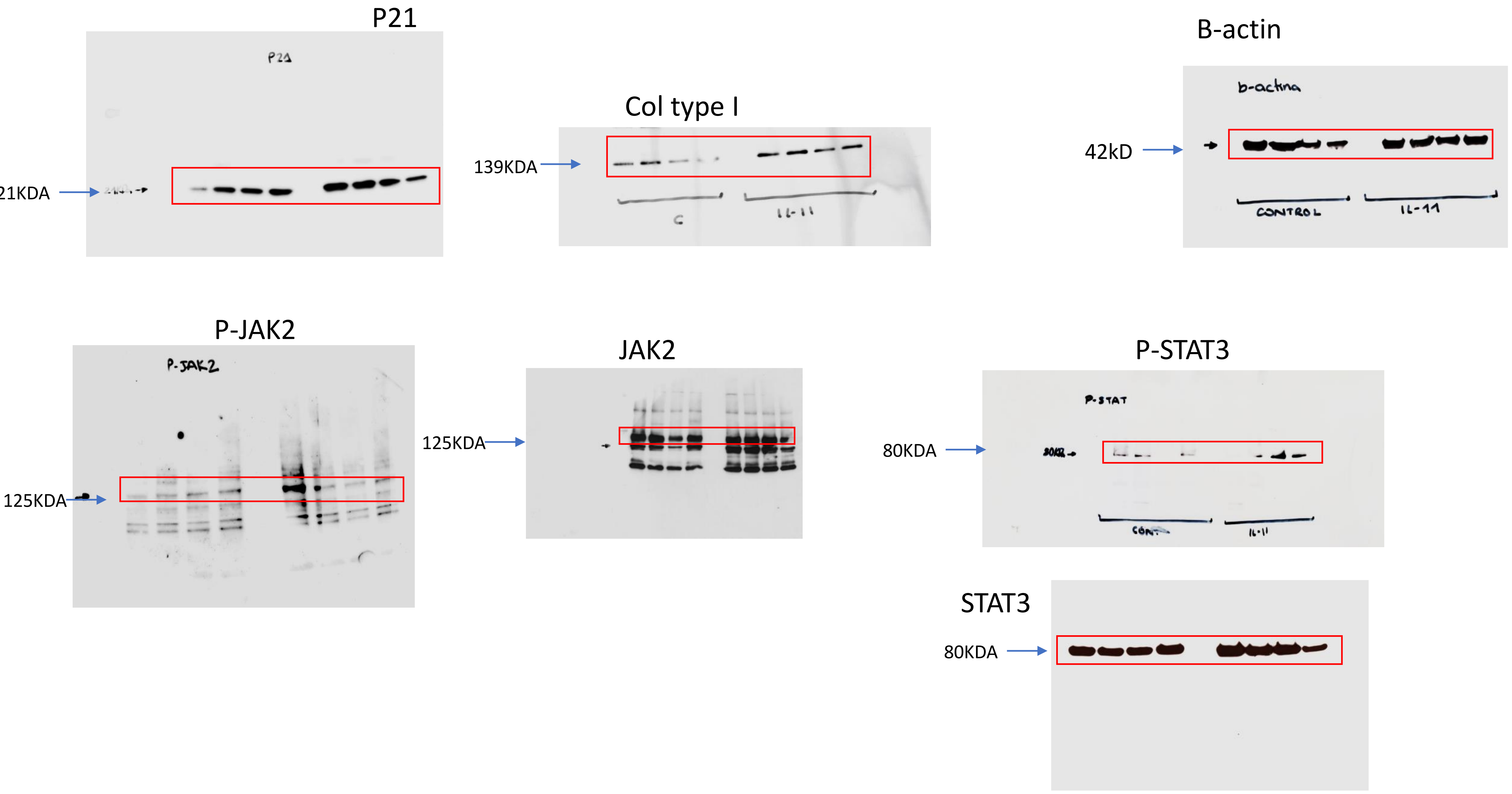

Supplementary figure S2

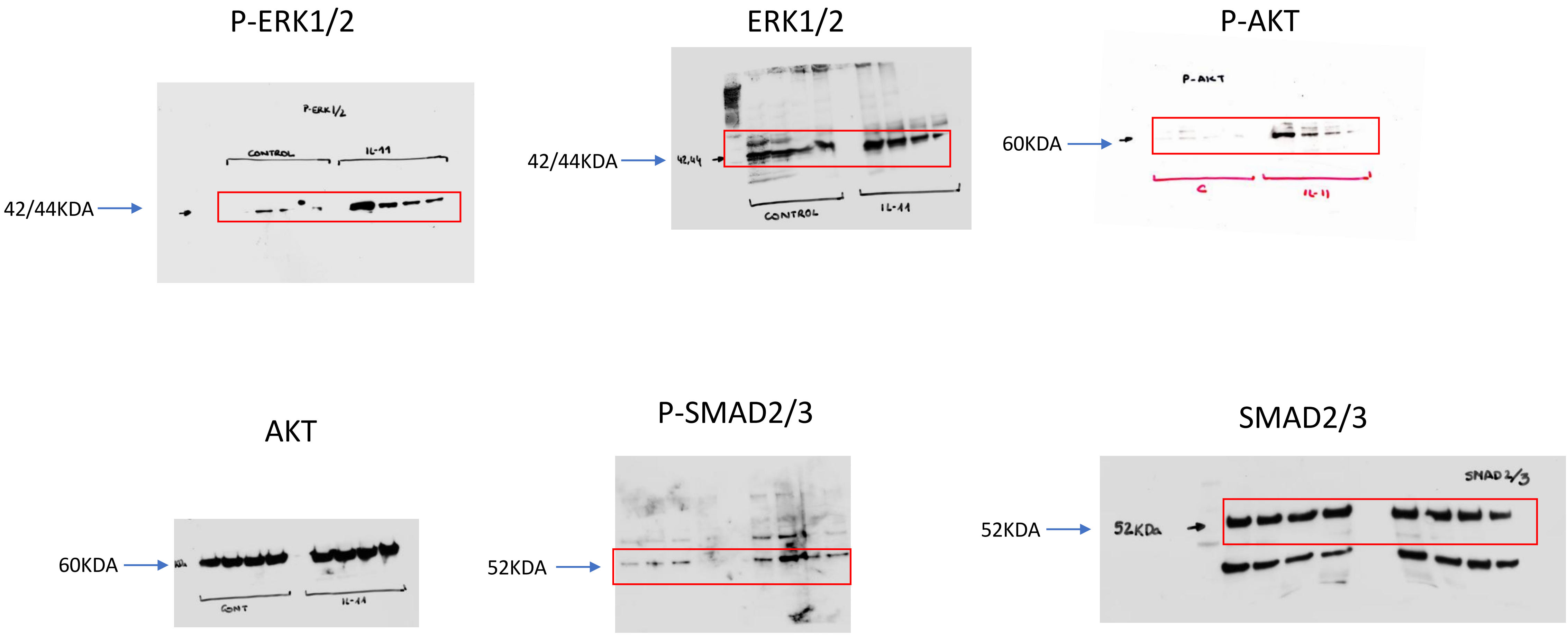

Supplementary figure S4

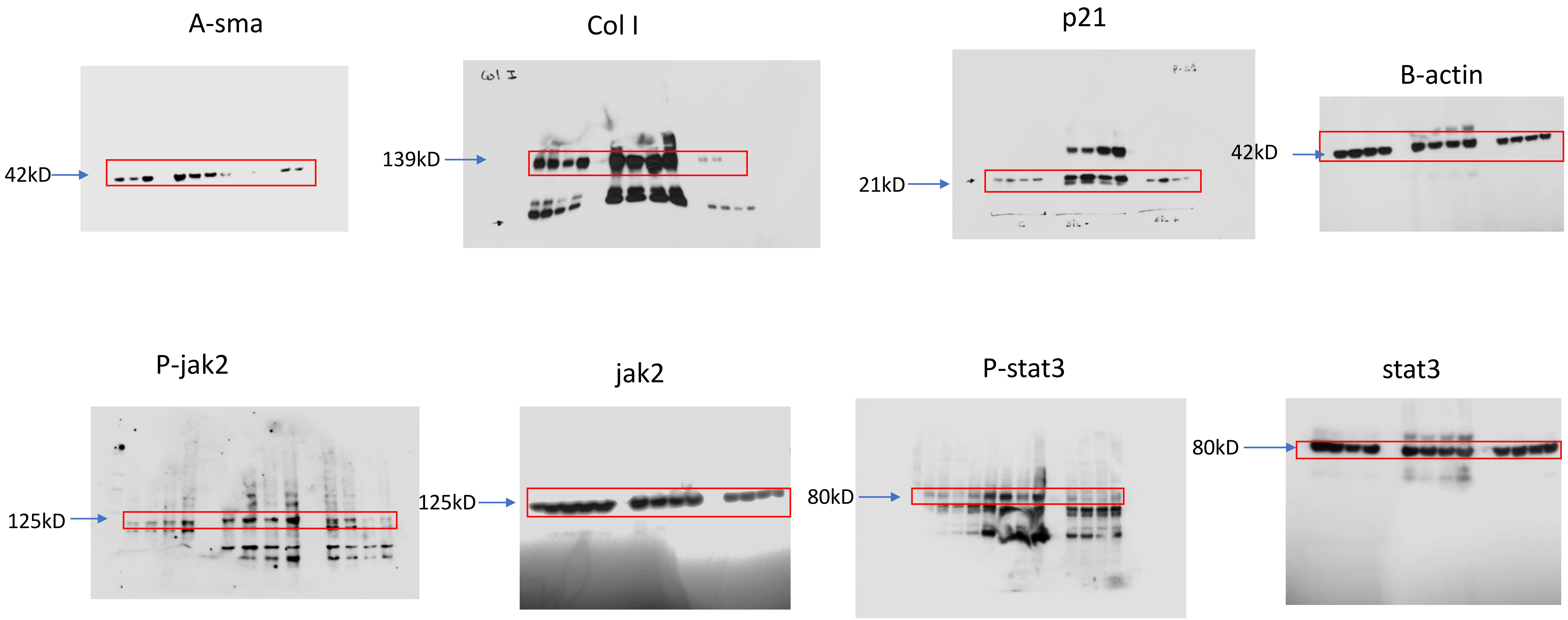

Supplementary figure S4

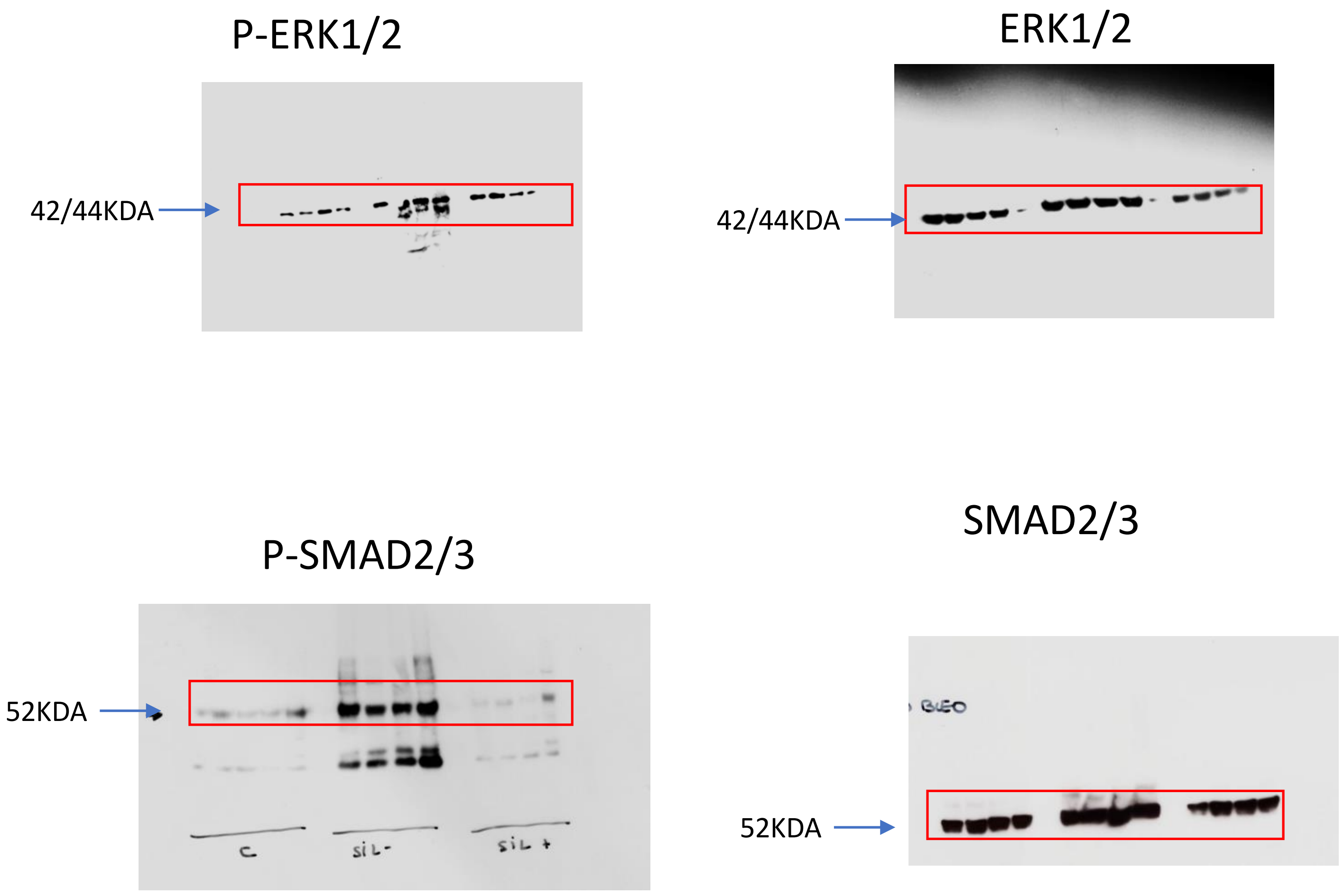

Supplement: Supplementary file 9 — Additional file 9. Supplementary figures. [file 12931_2022_2241_MOESM9_ESM.pdf]
